# Comparative metagenome-associated analysis of gut microbiota and antibiotic resistance genes in acute gastrointestinal injury patients with the risk of in-hospital mortality

**DOI:** 10.1128/msystems.01444-24

**Published:** 2025-02-27

**Authors:** Yunpeng Bai, Yali Hu, Xiangyin Chen, Linhui Hu, Kunyong Wu, Silin Liang, Jinshui Zheng, Michael G. Gänzle, Chunbo Chen

**Affiliations:** 1 Department of Pharmacy, Shenzhen People’s Hospital, The Second Clinical Medical College of Jinan University, The First Affiliated Hospital of Southern University of Science and Technology, Shenzhen, China; 2 College of Life Sciences, University of Chinese Academy of Sciences, Beijing, China; 3 Department of Critical Care Medicine, Shenzhen People’s Hospital, The Second Clinical Medical College of Jinan University, The First Affiliated Hospital of Southern University of Science and Technology, Shenzhen, China; 4 Department of Surgery Intensive Care Medicine, Maoming People’s Hospital, Maoming, China; 5 Department of Critical Care Medicine, Maoming People’s Hospital, Maoming, China; 6 Center of Scientific Research, Maoming People’s Hospital, Maoming, China; 7 Biological Resource Center of Maoming People’s Hospital, Maoming, China; 8 State Key Laboratory of Agricultural Microbiology, Hubei Hongshan Laboratory, Huazhong Agricultural University, Wuhan, China; 9 Hubei Key Laboratory of Agricultural Bioinformatics, College of Informatics, Huazhong Agricultural University, Wuhan, China; 10 Department of Agricultural, Food, and Nutritional Science, University of Alberta, 4-10 Ag/For Center, Edmonton, Canada; 11 Hubei University of Technology, College of Bioengineering and Food Science, Wuhan, China; Southern Medical University, Guangzhou, Guandong, China

**Keywords:** acute gastrointestinal injury, intensive care unit, metagenome-associated analysis, gut microbiota dysbiosis, antibiotic resistance genes, in-hospital mortality, critical illness

## Abstract

Acute gastrointestinal injury (AGI) is known for its poor long-term prognosis and the associated increase in mortality among intensive care unit (ICU) patients. As the role of the gut microbiome and resistome in AGI remains unclear, the present study aimed to explore the possible associations between dysbacteriosis and in-hospital mortality in ICU patients with gastrointestinal dysfunction. Fecal samples were collected from a prospective cohort of 210 ICU patients with AGI, and shotgun metagenomic sequencing was used to determine the taxonomic composition of gut microbiota and the differences of antibiotic resistance genes (ARGs) between the Death and Survival groups. Compared to the Survival group, patients in the Death group shifted from strict anaerobes to facultative anaerobes in the fecal microbial community, with more *Klebsiella* but less *Prevotella*. The co-occurrence patterns revealed that more ARG subtypes were enriched in microbial taxa in the Death group, especially for *Clostridium* and *Methanobrevibacter*. Furthermore, the ARG type had large area under the curve (AUCs) in receiver operating characteristic for predicting the disease severity, and a combined gut microbiota-ARG subtype classifiers showed better performance than either of them. Thus, comparative metagenome-associated analysis can help to obtain valuable information about gut microbiota and gene coding for antibiotic resistance in AGI patients.

**IMPORTANCE:**

A metagenomic-related strategy was conducted to obtain a highly valuable resource to improve understanding of intestinal microbiota dysbiosis and antibiotic resistance genes (ARGs) profiles. The results indicate that intestinal microbiota, including *Klebsiella* and *Prevotella*, changed dramatically in intensive care unit (ICU) patients with acute gastrointestinal injury (AGI). Due to longer ICU stays and receiving more antibiotic treatment, the types and correlations of ARGs in the Death group were significantly higher than those in the Survival group. The findings of this study are expected to expand our knowledge of gut microbiota and resistome profiles reflecting gastrointestinal status, accelerate the identification of disease biomarkers, and provide new insights into the prevention and treatment of AGI-related diseases.

## INTRODUCTION

Acute gastrointestinal injury (AGI) covers a broad range of symptoms in the gastrointestinal (GI) tract due to acute disease and surgery and is frequently observed in intensive care unit (ICU) patients (1). Approximately 62% of severe patients have a GI function injury, which is closely associated with digestive system dysfunction during hospitalization and with a worse clinical outcome (2). Furthermore, AGI may aggravate multiple organ failure, accelerate the progress of disease, and increase the mortality of critically ill patients (3). Although GI dysfunction is an important aspect of multiple organ dysfunction syndromes, the classification and diagnosis of AGI rely on clinical symptoms and signs, namely, gastric retention, diarrhea, intestinal obstruction, and intra-abdominal hypertension, rather than critical illness scoring tools (4). While some progress has been made in microbial indicators and biochemical markers reflecting GI function (5), there remains a lack of specific biomarkers and combined markers for clinical application. Therefore, discovering early AGI diagnostic biomarkers with high sensitivity and specificity has become a research hotspot in this field.

The intestine is the largest “bacterial reservoir” in the human body, with more than 1,000 species of bacteria, a total of approximately 10^14^ cells (6). The gut microbiota plays important roles for the host, such as host immunity, regulating intestinal endocrine functions, metabolizing drugs, producing beneficial compounds, and eliminating toxins that affect the host (7, 8). The beneficial symbiotic microbiota is typically dominant when the intestine is healthy, whereas the changes in the micro-ecological balance and pathogens may lead to intestinal disease if the beneficial microbiota is replaced (9, 10). As dysbiosis of the gut microbiota is closely related to many diseases, the clinical diagnosis based on the microbiome may be a breakthrough in some diseases, such as an early diagnosis of colorectal cancer (11). In a critical state, a variety of acute factors can cause rapid changes in the gut microbiota from symbiotic mode to pathogenic mode, including severe stress, intestinal ischemia/reperfusion injury, the use of broad-spectrum antibiotics, and destruction of barrier integrity in GI (12, 13). Such a “disease-promoting microbiome” or “pathological microbiota” associated with severe disease may lead to downstream inflammatory events in intestinal epithelial cells, which is the cause and consequence of GI injury and multiple organ dysfunction syndromes (14). However, the potential pathophysiological factors and relevance of GI injury in critically ill patients are still poorly understood.

The patient’s gut is not only a potential source of opportunistic pathogens but also a huge reservoir of antibiotic resistance genes (ARGs) (15). In recent years, rapid emergence of resistome has been stimulated by the overuse and misuse of antibiotics, further facilitating the selection of ARGs and increasing the possibility of the horizontal transmission of ARGs in the gut (16, 17). Antibiotics have a wide range of side effects on patients, and the harmful mechanisms may refer to immune cell toxicity, adverse drug reactions, disruption of the microbiome, mitochondrial toxicity, and the selection of drug-resistant organisms (18). For example, ICU patients were found to be susceptible to acquiring multidrug-resistant organisms due to their exposure to a large amount of antibiotic load prior to and during the ICU stay, a risk factor for isolating carbapenemase-resistant Enterobacterales (19, 20). As for microbiome diversity, both the critical illness itself and the frequent use of antimicrobials can contribute to the disruption of gut microbiota (21). Furthermore, a study on ventilator-associated pneumonia showed that the prolonged use of antibiotics cannot improve prognosis and may even increase the incidence of complications (22). Thus, it is necessary to investigate the influence of ICU stay on the microbiome and resistome in patients with AGI.

Metagenomics can comprehensively reveal microbial diversity and the interactions between microbial communities and the environment without relying on traditional cultivation techniques and species identification (23). Meanwhile, metagenomic sequencing combined with bioinformatics software has been used to better characterize the gut microbiota, including more accurately elucidating the composition, functions, gene coding, and potential influence on the host (24). In the present study, we report on the comparative metagenome-associated analyses using 210 fecal samples (80 deaths and 130 surviving ICU patients with AGI). The functional changes in the gut microbiota of ICU patients with AGI were determined using the human microbiome project (HMP) unified metabolic analysis network three (HUMAnN3) pathway analysis of fecal metagenomes. The present metagenomic study aims to clarify the changes in the gut microbiota, look for ARGs, and predict the risk of in-hospital mortality in ICU patients with AGI.

## MATERIALS AND METHODS

### Participants grouping and shotgun metagenomic sequencing

The research has been performed according to the World Medical Association Declaration of Helsinki, and the local ethics committee of Maoming People’s Hospital approved the study with No. PJKY2020MI-149–01. The prospective cohort of patients was consecutively recruited in the adult ICU, surgical ICU, and cardiac surgery ICU of the hospital from April 2021 to February 2022, and written informed consents were also obtained from the patients or their relatives before ICU admission. Participants were enrolled due to the varying degrees of GI dysfunctions and were excluded with any of the following criteria: (i) the age of the participant was less than 18 years old; (ii) the participant admitted to ICU due to end-stage chronic diseases; (iii) the participants received systemic antibiotic treatment within 48 hours before ICU admission; or (iv) the participants died within 24 hours after ICU admission. Demographics and clinical indicators were recorded at ICU admission, and Acute Physiology and Chronic Health Evaluation (APACHE) II scores were assessed as well.

Fecal samples or rectal swabs (if stool was unavailable) were collected during ICU period with fecal protective solution kit and immediately frozen at −80°C, which were obtained from Biological Resource Center of Maoming People’s Hospital. All samples were then transported to BGI-Shenzhen with dry ice for extraction. The total DNA was automatically extracted using magnetic bead Magen kit, and the quantity and quality of DNA were assessed by DNA Qubit and AGE electrophoresis. After detection, all DNA samples were used to construct the library that was examined by Agilent 2100 bioanalyzer. Paired-end metagenomic sequencing was then performed using the DNBSEQ-T1 platform (insert size, 350 bp; read length, 100 bp).

### Bioinformatic analysis of metagenomic sequencing

Adaptor and low-quality reads (≤70) were discarded from the raw reads via the default mode of fastp, and the remaining reads were filtered to eliminate host DNA based on the human genome reference (hg38) using the “very sensitive” mode of bowtie2 (25). On average, 7.69 Gb of high-quality non-host data were obtained per sample in the research. HUMAnN3 was applied to obtain the compositions of the microbial communities and the abundance of functional pathways (26). Metagenomic phylogenetic analysis (MetaPhlAn3) embedded in HUMAnN3, and chocophlan pan-genome databases were used to obtain species composition information, and MinPath (27), Diamond (28), UniRef (29), and MetaCyc databases (30) were used to annotate gene families, functions, and pathways. Finally, gene-family abundance, pathway abundance, and pathway coverage profiles were generated from HUMAnN3 algorithm. Key parameter for HUMAnN3 included the following: humann --input <input.fastq> --output <output_dir> --metaphlan-options <metaphlan_options>. Furthermore, abundance outputs were normalized with the humann-renorm-table command.

The retrieved sequences were used to search for ARGs following ARGs-OAP v2.0 pipeline with default parameters (31). The Structured Antibiotic Resistance Gene (SARG, version 3.2) database was used to annotate ARG types (antibiotic class) and within that class subtypes (e.g., a subtype having >80% identical aligned bases based on hidden Markov models). The abundances of ARGs were determined as copy number (one read per copy) per million read by normalizing the number of ARGs-containing reads against that of total reads. Unclassified ARG types and subtypes were excluded in this study.

### Statistical analyses

In order to understand the differences in microbiota compositions, metabolic pathway, and ARG diversities among samples, we performed alpha diversity and beta diversity analyses. α-Diversity was calculated through the Shannon index depending on the abundance profile, and permutational multivariate analysis of variance was used to assess the differences. β-Diversity was estimated using Bray-Curtis dissimilarity, and principal coordinates analysis (PCoA) on Bray-Curtis distance was performed to obtain visual representations. The analysis of α-diversity and β-diversity was performed using the package “vegan.” All the above statistical analyses and visualizations were performed in R (v4.4.4).

Linear discriminant analysis (LDA) effect size (LEfSe) analysis was used to identify the differential abundance of taxa and functional pathways between groups. This method used the non-parametric factorial Kruskal-Wallis sum-rank test to investigate features with significant differential abundance, and LDA was then used to obtain the effect size of each feature. The LEfSe analysis was done using a Galaxy computational tool (http://huttenhower.sph.harvard.edu/galaxy/). Differences between groups were also assessed using two-tailed Welch’s *t*-tests using STAMP (v2.1.3). In addition, correlation heatmaps were conducted to investigate the relationship between differential gut microbiota and differential pathways based on the results of LEfSe and STAMP.

Meanwhile, the differential ARGs and ARG types were screened out after error correction and normalization based on the coefficient and *q* values (*P* values corrected by the false discovery rate(FDR) via the Benjamini-Hochberg (BH) procedure) <0.05 as the judgment criteria for significant differences. The R package corr.test analysis was used to compute the Spearman correlation coefficients of differential ARG types in ICU patients with AGI. Furthermore, network and heatmap analyses were used to explore the co-occurrence of ARG subtypes and microbial taxa based on Spearman correlation analysis (*P* value < 0.05). Correlation coefficients and significant *P* values were computed using R software.

### Identification of microbiota and ARG markers for predicting in-hospital mortality

To identify microbial and ARG markers distinguishing the Death and Survival groups, we performed classification models based on species, ARG types, and subtypes using the random forest (RF) model. Each model was validated using five trials of the tenfold cross-validation, and the accuracy of the models was assessed using receiver operating characteristic (ROC) curves and confusion matrix. The RF analysis was conducted in two steps. In the first step, separate models were constructed using species and ARG profiles independently. Fifty-three bacterial species with at least 0.5% relative abundance in at least 50% of all samples in both groups were selected for further analysis in the microbial species model. All prefiltered features were included in the RF function to compute “mean decrease accuracy” and “mean decrease gini.” The top 13 important features were used for the final model construction. Considering the imbalance in sample sizes between the Death and Survival groups, the separate pre-filtering process in each group was performed on the strains with a relative abundance of 0.5% in at least 50% of the samples, which was further merged as the feature set for model comparison. In the second step, a combined model was built using features selected from the separate models (microbiota and ARGs). All analyses were performed using the R software (version 4.3.0) with the “randomForest” package, and ROC curves were plotted using the “pROC” package.

## RESULTS

### Microbiome comparison in ICU patients with AGI between Death and Survival groups

A total of 210 ICU patients with GI injury were enrolled in this study with a mean age of 64.1 years, including 80 patients (38.1%) in the Death group and 130 patients (61.9%) in the Survival group based on clinical outcomes. The patients’ clinical demographics are shown in Table 1. Notably, the days of ICU stay and the APACHE II scores were significantly higher in the Death group than the Survival group (*P* < 0.05), which also meant that patients could receive more antibiotic treatment with the increase of ICU stay. In total, 3,901,338 genes were identified via the metagenome-associated analysis, and 837 microbial species were identified after removing the unannotated species, which represented 81 families and 233 genera (Table S1). The top 10 abundance stacking maps at the genus and species levels are shown in Fig. S1A and B, and *Bacteroides*, *Escherichia*, *Enterococcus*, and *Klebsiella* were the most dominant genera in both groups. Compared with the Survival group, the Death group showed an increased abundance of *Escherichia coli* and *Klebsiella pneumoniae*, along with decreased abundance of *Bacteroides vulgatus*), *Faecalibacterium prausnitzii*, and *Prevotella copri*. In addition, relative abundance correlation analysis of the top 10 genera showed that *Escherichia* was significantly correlated with *Klebsiella* in the Death group (Fig. S1C), whereas *Escherichia* and *Klebsiella* were negatively correlated with *Enterococcus* in the Survival group (Fig. S1D).

**TABLE 1:** Clinical and demographic characteristics of ICU patients

Variables	Total (*n* = 210)	Death (*n* = 80)	Survival (*n* = 130)	*P* value
Age, years	64.1 ± 16.4	71.8 ± 14.7	56.1 ± 15.2	0.000
Male, *n* (%)	134 (63.8)	53	81	0.000
ICU stay, days	10.9 ± 11.5	12.9 ± 12.3	9.4 ± 10.8	0.048
ICU type, *n* (%)				<0.001
Adult ICU	55 (26.2)	32	23	
Surgical ICU	115 (54.8)	47	68	
Cardiac surgery ICU	40 (19.0)	1	39	
APACHE II score	18.8 ± 8.7	25.3 ± 7.8	14.6 ± 6.6	0.000

The Shannon index and PCoA based on the Bray-Curtis distance were separately performed to compare the α-diversity within a sample (Fig. S2) and β-diversity among samples (Fig. S3) at the family, genus, species, and functional pathway levels. As a result, no significant difference was observed between the Death and Survival groups with respect to the α-diversity at the family, genus, or species level. Whereas, the α-diversity of the gut microbiota functional pathways in the Death group was significantly higher than that in the Survival group (*P* < 0.001). In the β-diversity analysis, significant differences in the microbiota composition (family, genus, and species) and functional pathway were observed between the Death and Survival groups (*P* < 0.001).

**Fig 1:**
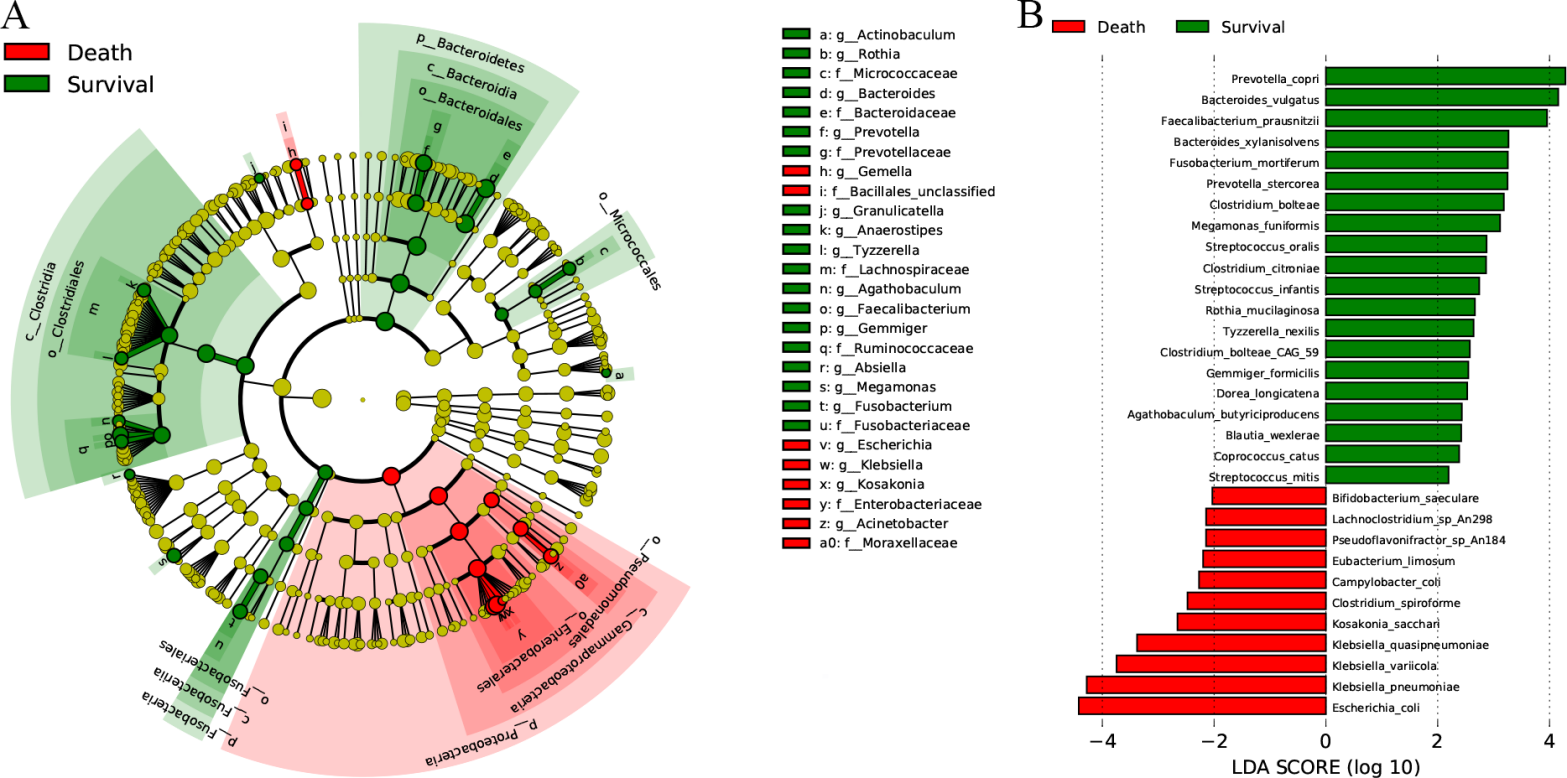
LEfSe analysis of the variations in the composition of the fecal microbiota in the Death and Survival groups. (**A**) Cladogram at the family and genus levels, LDA > 2.0, *P* < 0.05. (**B**) LEfSe analysis at the species levels, LDA > 2.5, *P* < 0.05. Red indicates the enriched flora in the Death group, while green indicates the enriched flora in the Survival group.

To investigate the differentially abundant taxa, LEfSe analysis and Welch’s *t*-test were carried out on the fecal microbiota composition and functional pathways in the Death and Survival groups, respectively. In LEfSe analysis, 9 bacterial families and 18 genera (Fig. 1A) had distinct relative abundances between the two groups (LDA score >2.0, *P* < 0.05). A decreased abundance of gut microbiota was observed in the Death group, which included *Bacteroides*, *Prevotella*, *Faecalibacterium*, *Megamonas*, *Fusobacterium*, *Gemmiger*, *Actinobaculum*, *Anaerostipes*, *Granulicatella*, *Tyzzerella*, *Rothia*, *Absiella*, and *Agathobaculum*, while the increased abundance in the Death group was *Gemella*, *Kosakonia, Acinetobacter*, *Escherichia*, and *Klebsiella*. In the same way, there were 31 bacterial species (Fig. 1B, LDA score >2.5, *P* < 0.05) and 143 bacterial functional pathways (Fig. S4A; Table S2, LDA score >2.5, *P* < 0.05) with distinct relative enrichment between the two groups.

As for Welch’s *t*-test, 5 bacterial families (Fig. S5A) and 10 bacterial genera (Fig. S5B) showed distinct relative abundances between the two groups (*P* < 0.05). In the Death group, it was observed that *Agathobaculum*, *Dorea*, *Faecalibacterium*, *Bacteroides, Gemmiger*, *Prevotella*, *Roseburia*, and *Streptococcus* were less abundant, while more abundant taxa included *Escherichia* and *Klebsiella*. In the same way, 30 bacterial species (Fig. S5C) and 99 bacterial functional pathways (Fig. S4B; Table S2) showed distinct relative enrichment between the two groups (*p_corrected* < 0.05). According to the above two differential analyses, the facultative anaerobes, such as *Enterobacteriaceae*, were enriched in the Death group. At the species level, *K. pneumoniae*, *E. coli*, *Klebsiella variicola*, and *Klebsiella quasipneumoniae* were increased in the Death group, while *P. copri* and *F. prausnitzii* were decreased in the Death group. Since age is a critical factor influencing patient outcomes, a more age-homogeneous subgroup (from 30 to 70 years old, *P* > 0.05 between the Death and Survival groups) was established and performed for differential analysis using STAMP method (Fig. S6). After comparison, the genera of *Klebsiella* and *Prevotella* showed consistent trends with the original grouping without considering the age factor. However, the genus *Escherichia* (including *E. coli*) was not enriched in the subset of the Death group, indicating a more significant correlation between *E. coli* and the age factor.

**Fig 2:**
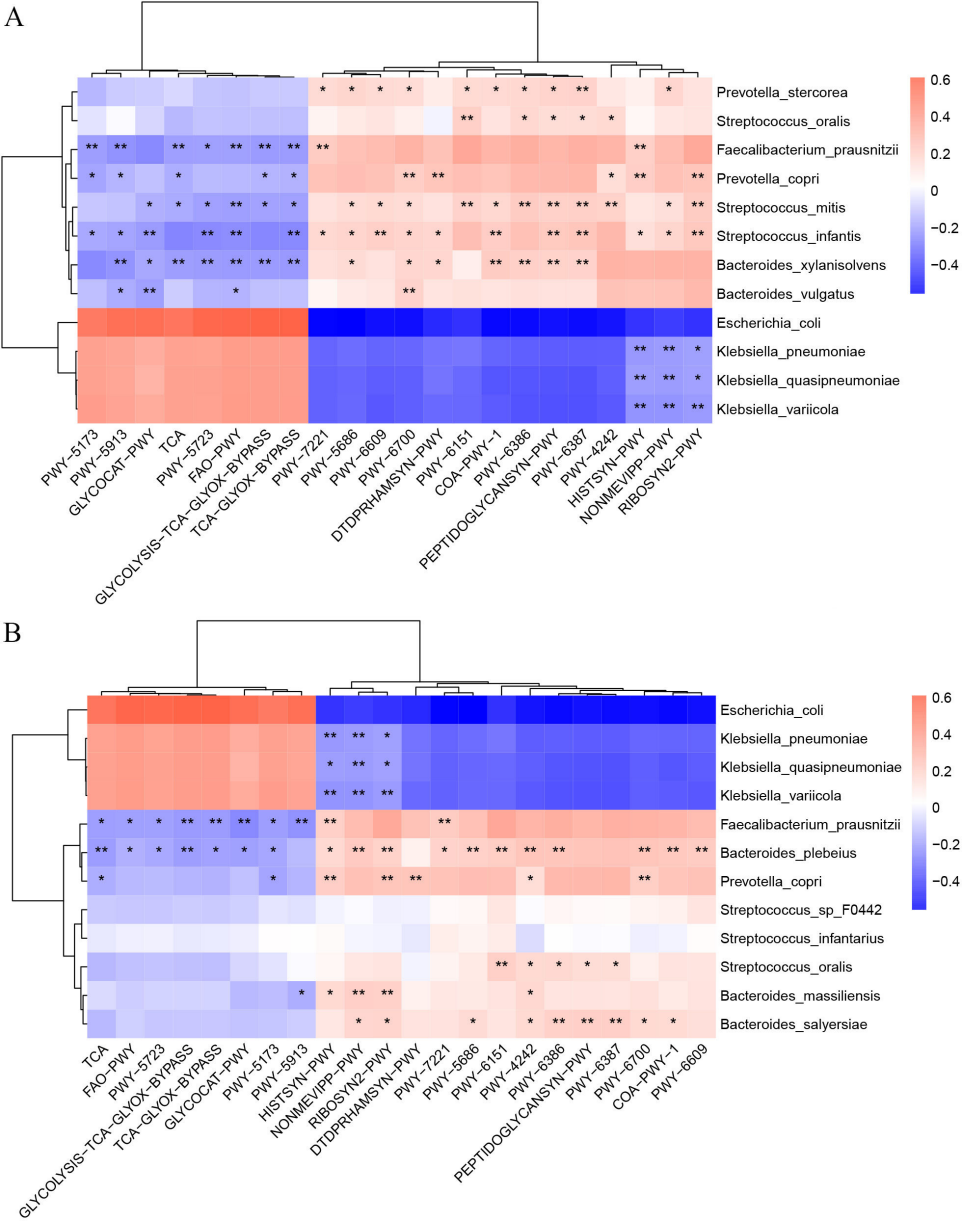
Heatmaps showing the association between the differential intestinal microbiota of species and differential pathways in all ICU patients with AGI. (**A**) LEfSe analysis. (**B**) STAMP analysis. Single asterisks indicate statistical significance based on Spearman correlation with *P*  <  0.05, and double asterisks indicate statistical significance with *P* < 0.01. Cor. coef., correlation coefficient.

In order to compare the functional pathways of gut microbiota in ICU patients with AGI, the pathway differences were further investigated between the two groups through LEfSe and STAMP analysis (Table S2). Totally, 14 pathways in the Death group and 47 pathways in the Survival group were identified based on the LEfSe analysis (LDA > 2.7). The enrichments in the Death group were fatty acid elongation-saturated, superpathway of glyoxylate bypass and tricarboxylic acid (TCA), stearate biosynthesis II, TCA and glyoxylate bypass, Rubisco shunt, among others (LDA > 2.7), while the superpathways of coenzyme A biosynthesis III, UMP biosynthesis I, flavin biosynthesis I, methylerythritol phosphate pathway I, among others were enriched in the Survival group (LDA >3.0). Similarly, the STAMP results showed that 99 differential pathways were detected (including 64 pathways in the Death group), which was slightly different from the LEfSe analysis with variable proportions. The pathways of flavin biosynthesis I (0.47% ± 0.23% vs 0.69% ± 0.29% in the Death and Survival group, respectively, *P* = 1.30E-5), peptidoglycan biosynthesis I (0.77% ± 0.23% vs 0.96% ± 0.27%, *P* = 1.21E-4), methylerythritol phosphate pathway I (0.63% ± 0.26% vs 0.84% ± 0.31%, *P* = 2.36E-4) among others, were significantly different between the Death and Survival groups. Furthermore, related analyses between 12 differential intestinal species and 22 differential functional pathways were performed according to the results of LEfSe and STAMP (Fig. 2). The relevant heatmaps indicated that changes of functional pathways can be attributed to disturbances in gut microbiota involving *Klebsiella*, *Bacteroides*, *Faecalibacterium*, *Streptococcus*, *Prevotella*, and others.

### Resistome comparison in ICU patients with AGI between Death and Survival groups

As for ARGs, a total of 9,891 of ARGs (27 types and 603 subtypes of antibiotic resistance) were detected in the 210 samples (Table S3), including antibacterial fatty acid, aminoglycoside, bacitracin, β-lactam, bicyclomycin, bleomycin, chloramphenicol, defensin, florfenicol, fosfomycin, fusidic-acid, factumycin, macrolide lincosamide streptogramin (MLS), multidrug, mupirocin, novobiocin, other peptide antibiotics, polymyxin, pleuromutilin tiamulin, quinolone, rifamycin, streptothricin, sulfonamide, tetracycline, trimethoprim, tunicamycin, and vancomycin. A newly identified ARG (named *capO*) is also listed in Table S3, belonging to the type of chlorphenicol and the antibiotic resistance mechanism of enzymatic inactivation (32). As shown in Fig. S7A, the top five dominant ARG types were tetracycline*,* MLS, multidrug, β-lactam, and aminoglycoside in both groups, while the three lowest abundance ARG types (values < 1/100,000) were tunicamycin, fusidic-acid, and factumycin. In addition, the mechanisms of antibiotic resistance are separately shown in Fig. S7B (Death group) and Fig. S7C (Survival group). As shown in the figures, the resistome abundances were mainly contributed by enzymatic inactivation, antibiotic target alteration, antibiotic target protection, and efflux pump.

**Fig 3:**
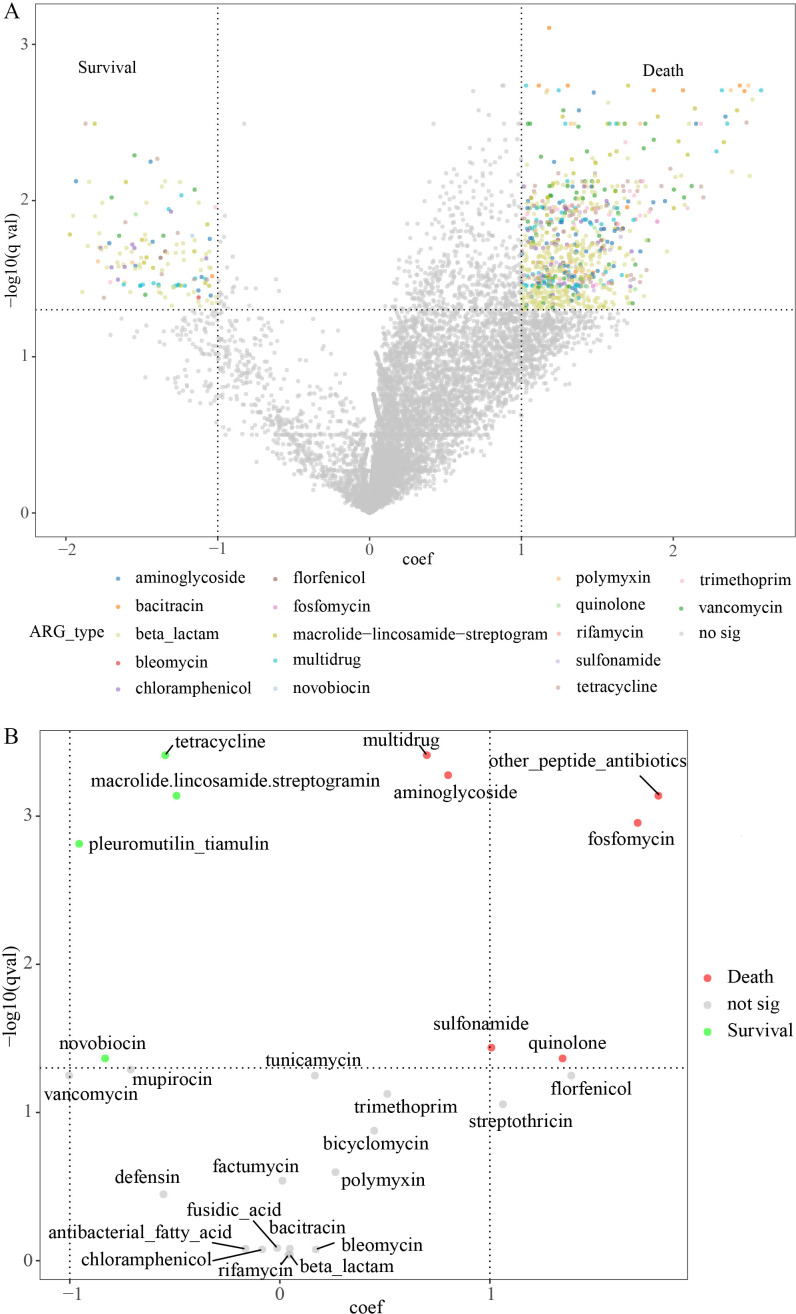
Comparative ARGs in AGI patients between the Death and Survival groups. (**A**) The volcano plot of the differential ARGs in both groups. Each point represents an ARG, each color indicates a type of ARG, and the nonsignificant ARGs are marked as gray. (**B**) The scatter plot of the differential ARG types in both groups.

The differential comparison of ARGs between the Death and Survival groups is shown in Fig. S8, which indicates the higher α-diversity in the Death group and significant differences in ARG composition in the β-diversity between the two groups (*P* < 0.001). Furthermore, a volcano plot was used to display the difference in quantitative values of ARGs (Fig. 3A), indicating that more types of ARGs were enriched in the Death group. As shown in the scatter plots of ARG types (Fig. 3B; Table S4), the enriched ARG types in the Death group included aminoglycoside, multidrug, fosfomycin, sulfonamide, quinolone, and other peptide antibiotics, whereas the enriched ARG types in the Survival group were tetracycline, MLS, pleuromutilin tiamulin, and novobiocin. The relative abundance correlation analyses were performed in the Death and Survival groups to investigate the relationships of the enriched ARG types (Fig. S9). The results showed that multidrug and tetracycline, multidrug and MLS, other peptide antibiotics, and MLS were all significantly negatively correlated in the Death group. Positive correlations were also observed in both groups, including multidrug and other peptide antibiotics, tetracycline, and pleuromutilin tiamulin.

Next, *t*-test analysis was performed to compare the relative abundances of subtypes with high values in the aforementioned differential types between the two groups and determine the significant ARG subtypes (Table S5) involved in the changes (Fig. S10). Compared with the Survival group, the increased abundances of ARG subtypes in the Death group were *aph(3'')-Ib*, *aph (6)-Id*, *aac(6')-Ib-cr* in aminoglycoside type (*P* < 0.01), *mdtK*, *msbA*, *mdtL*, *E. coli mdfA*, *emrD*, *mdtH*, *acrF*, *mdtE*, *tolC*, *emrA*, *emrB, acrE, MexB, sdeY*, *K. pneumoniae acrA*, *MexE*, and *MuxB* in the multidrug type (*P* < 0.001); *FosA family* in the fosfomycin type (*P* < 0.001); *microcin efflux pumu gene yojI* in other peptide antibiotics type (*P* < 0.001); *QnrB family* in the quinolone type (*P* < 0.05); and *sul1* in the sulfonamide type (*P* < 0.001). Meanwhile, some ARG subtypes with decreased abundances were observed in the Death group, including *tet*(*Q*), *tet*(*O*), *tet*(*X*), *tet*(32), *tet*(37), and *tetB*(*P*) in the tetracycline type (*P* < 0.05); *erm(F)*, *mel, LlmA 23S ribosomal RNA methyltransferase*, *RlmA*(*II*), and *erm*(35) in the MLS type (*P* < 0.05); and *TaeA* in the pleuromutilin tiamulin type (*P* < 0.001). Therefore, the relative abundance changes of certain ARG types (e.g., aminoglycoside, multidrug, MLS, and tetracycline) were associated with alterations in some subtypes. For fosfomycin, other peptide antibiotics, pleuromutilin tiamulin, quinolone, and sulfonamide ARGs, only one subtype corresponded to the change in the relative abundance of the ARG types.

### Co-occurrence patterns and prediction of in-hospital mortality for ARGs and microbial taxa in ICU patients with AGI

**Fig 4:**
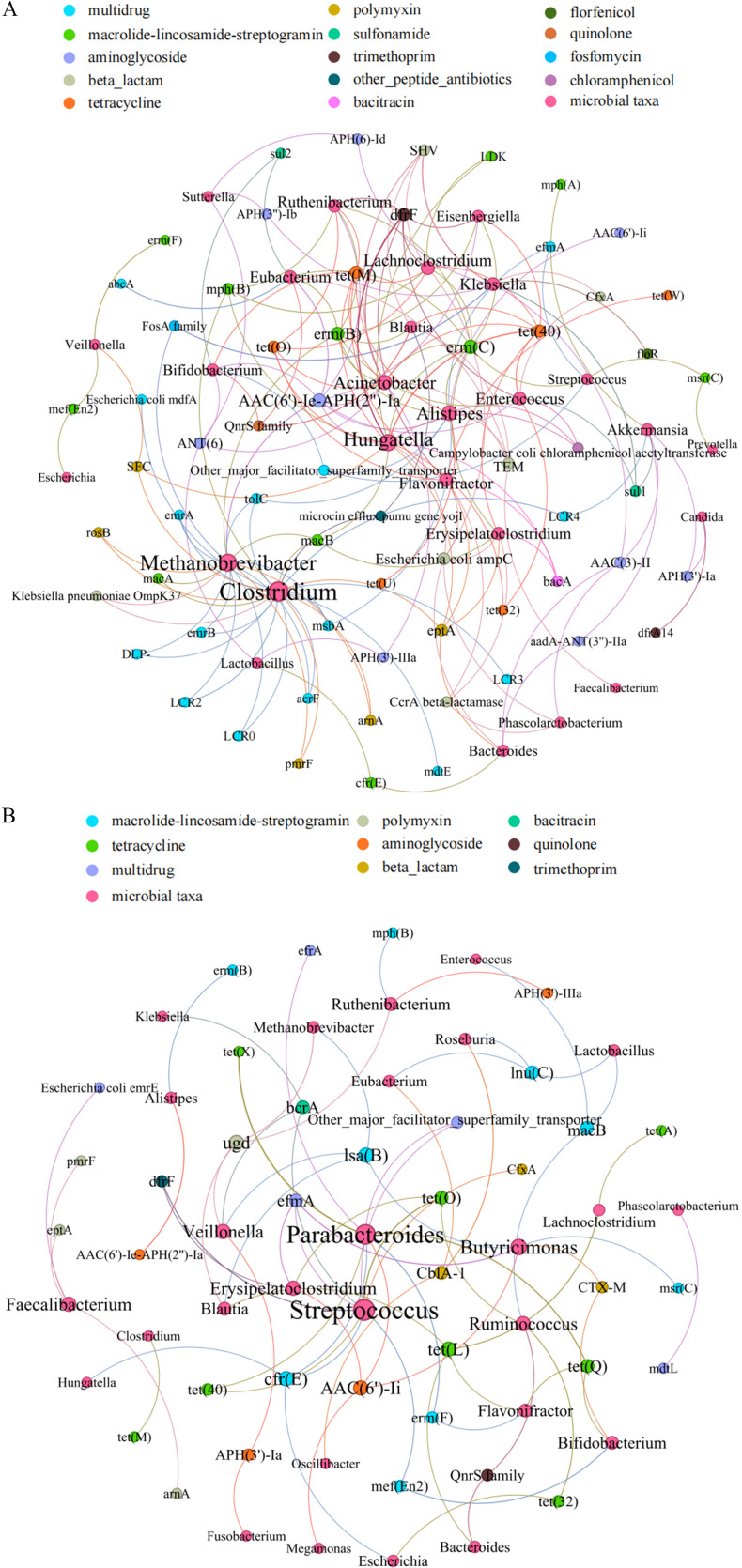
Network analysis revealing the co-occurrence patterns between ARG subtypes and microbial taxa. (**A**) The Death group. (**B**) The Survival group. The nodes were colored in accordance with the ARG subtypes and microbial taxa. A connection represents a significant correlation. Edges weighted according to the correlation coefficient and node size weighted according to the number of connections.

In studying the correlation between microbial genus and ARG subtype, network analysis was conducted to determine detailed relationships with the significant correlations. A total of 30 genera and 70 ARG subtypes with high abundances were selected for the analysis, and the results showed significant differences in the potential primary bacteria carrying ARGs between the two groups. Furthermore, the co-occurrence results (Fig. 4A) showed that the potential hosts of 61 ARG subtypes belonged to 26 bacterial genera in the Death group. *Clostridium* and *Methanobrevibacter* were the main bacterial genera associated with ARGs, with potential hosts for 22 and 17 ARG subtypes, respectively. *Hungatella* and *Klebsiella* also carried 15 and 8 ARG subtypes, respectively. However, 27 bacterial genera were potential hosts for 36 ARG subtypes in the Survival group (Fig. 4B). *Streptococcus* and *Parabacteroides* were the main bacterial genera associated with ARGs, with potential hosts for nine and eight ARG subtypes. In addition, *Klebsiella* might only carry one ARG subtype.

**Fig 5:**
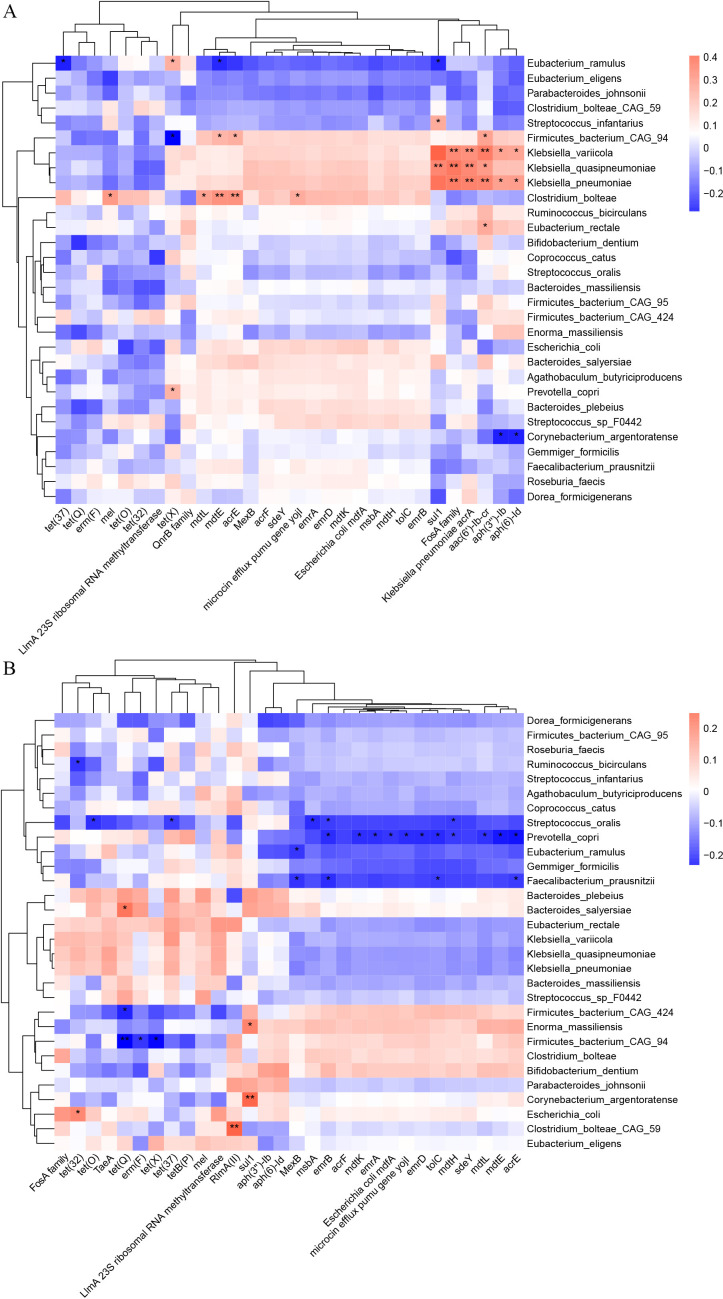
Heatmaps of Spearman correlation coefficients between disease-severity-related ARG subtypes and microbial species. (**A**) The Death group. (**B**) The Survival group. The color in the heatmap represents the correlation coefficients estimated by Spearman correlation analysis. Single asterisks indicate *P* values < 0.05, while double asterisks indicate *P* values < 0.01.

To investigate the co-occurrence patterns between gut microbiota and the disease severity-related ARGs, a correlation analysis was also performed in accordance with the relative abundance of ARG subtypes and gut microbiota composition at the species level. Here, 30 differential species and 30 differential ARG subtypes with high abundances were separately selected in the two groups for heatmap analysis. In the Death group (Fig. 5A), the results showed that *sul1*, *FosA family*, *K. pneumoniae acrA*, *aac(6’)-Ib-cr*, *aph(3’’)-Ib*, and *aph(6)-Id* were positively related to *Klebsiella* with a higher proportion of hospital-acquired pneumonia. Furthermore, *mel*, *mdtL*, *mdtE*, *acrE*, and *microcin efflux pumu gene yojI* were also positively related to *Enterocloster bolteae*, which might be a marker for autism or liver transplant rejection in rats (33). However, in the Survival group (Fig. 5B), the obvious alterations were significantly negative correlations between two microbial species and multiple ARG subtypes, including *S. infantarius* with *tet(O)*, *tet(37)*, *msbA*, *emrB*, *mdtH*, *P. copri* with *emrB*, *mdtK*, *emrA*, *E. coli mdfA*, *microcin efflux pumu gene yojI*, *emrD*, *tolC*, *mdtH*, *mdtL*, *mdtE*, and *acrE*.

**Fig 6:**
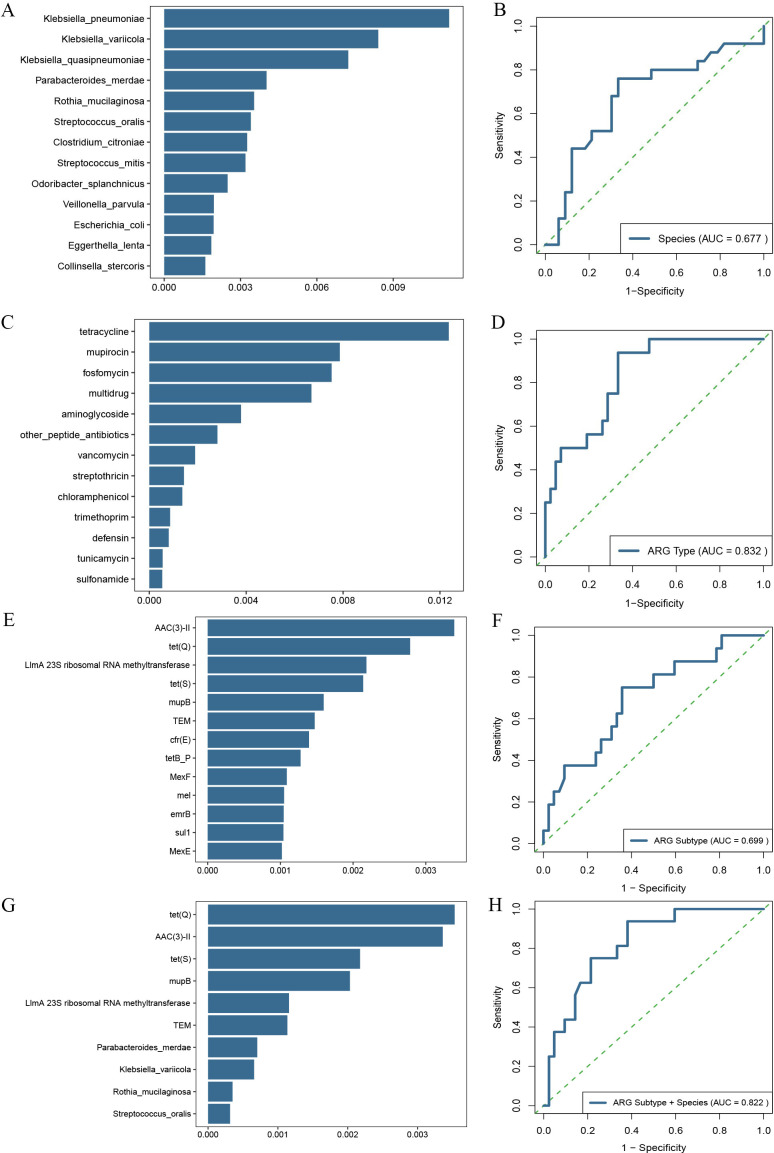
Diagnostic potential of gut microbial and ARG markers for the risk of in-hospital mortality in ICU patients with AGI. (**A**) The top 13 species in terms of importance. (**B**) ROC curve of species for the discriminative ability between two groups. (**C**) The top 13 ARG types in terms of importance. (**D**) ROC curve of ARG type between two groups. (**E**) The top 13 ARG subtypes in terms of importance. (**F**) ROC curve of ARG subtype between two groups. (**G**) The top 10 features for the combination of species and ARG subtypes in terms of importance. (**H**) ROC curve of the combination of species and ARG subtype between the two groups.

Next, an RF classifier model in the discovery cohort was constructed to evaluate the potential markers, enabling the assessment of the risk of in-hospital mortality in ICU patients with AGI. The top 13 species, in terms of importance, were selected as the optimal microbial marker set by a 10-fold cross-validation of the RF model (Fig. 6A). The AUC of the ROC curve was 0.677 with a 95% CI of 0.5546–0.8046 between the Death and Survival groups (*P* < 0.05) in the validation cohort (Fig. 6B). As for the comparison model with separate pre-filter process in each group, the results (Fig. S11) showed some changes in the marker selection, including the alteration of top species and the decease of ROC value. In addition, the confusion matrix as the additional performance metrics also showed that F1 score had a relatively lower value in the separate pre-filter process for each group (Fig. S12). Thus, the pre-filter process for both groups was selected for the model construction and the following RF analysis. To further identify the antibiotic resistance characteristics associated with the risk of in-hospital mortality, RF classification analysis was also performed at the ARG type and subtype levels. At the ARG type level, the RF classifier identified the top five ARG types of tetracycline, mupirocin, fosfomycin, multidrug, and aminoglycoside between the Death and Survival groups (Fig. 6C), and the AUC value of 0.832 was obtained for the characteristics of in-hospital mortality (Fig. 6D). At the ARG subtype level, the RF classifiers achieved AUC values of 0.699 (Fig. 6F), and the identified five top characteristic ARG subtypes were *aac (3)-II*, *tet(Q)*, *LImA 23S ribosomal RNA methyltransferase, tet(S)*, and *mupB* (Fig. 6E). Finally, the combination of microbial species and ARG subtypes was plotted for the ROC curve, and the top 10 features (*tet[Q]*, *aac[3]-II*, *tet[S]*, *mupB*, *LImA 23S ribosomal RNA methyltransferase*, *TEM*, *P. merdae*, *K. variicola*, *R. mucilaginosa*, and *S. oralis*) and AUC value of 0.822 were separately shown in Fig. 6G and H.

## DISCUSSION

The present study aimed to generate high-quality intestinal microbiota data sets from ICU patients with AGI and to compare the alterations in the resistome profiles and gut microbiota with in-hospital mortality. GI dysfunction is relatively common in ICU patients due to the vulnerability of their organs, suggesting the critical condition and disease severity of the patients (34). The intestinal microbiome of severely ill patients can change sharply within 6 hours after the occurrence of critical illness (35), and dysbiosis of gut microbiota can also be characterized by a loss of richness and diversity (36). Due to the increased permeability of the intestinal epithelial barrier, such pathogens can translocate and aggravate inflammation, which is an important cause of secondary infection and multiple organ dysfunction syndromes in critically ill patients (37, 38). Thus, endogenous gut bacteria and gene markers may be associated with the morbidity and mortality of acute illness and serve as new mortality predictors in critically ill patients (39).

Shotgun metagenomic sequencing can sequence the full complement of genes present in a microbiome and provide information about the abundance of genes at all taxonomical levels and functional pathways (40). In the study, a total of 837 microbial species (representing 81 families and 233 genera) and 9,891 ARGs (belonging to 27 types and 603 subtypes) were detected in 210 ICU patients with AGI, all of whom received antibiotic treatment during their hospitalization. Based on different clinical outcomes, the two groups (Death vs Survival) revealed different microbial compositions, functional pathways, and ARG profiles, which could provide an important source of diagnostic and prognostic biomarkers. Obviously, the shift was observed in the Death group, from strict anaerobes to facultative anaerobes (particularly *Enterobacteriaceae*), whose dysbiosis was quite comparable to the changes in inflammatory bowel disease (IBD) (41, 42) or antibiotic treatment (43, 44). Furthermore, the increased pathways of microbial diversity in the Death group indicated that the functions of gut microbiota might be related to the severity of the disease (45).

Compared with the Survival group, significant dysbiosis of the gut microbiota was correlated with the risk of in-hospital mortality in the Death group, particularly the increase in *Escherichia* (*E. coli*) and *Klebsiella* abundance as well as the decrease in *Bacteroides*, *Faecalibacterium* (*F. prausnitzii*), and *Prevotella* (*P. copri*) abundance. *E. coli* is the main aerobic organism in the intestine (46). Various species of *E. coli* could severely resist the commonly administered antibiotics and increase the incidence of carbapenem-resistant in ICU patients (47, 48). *Klebsiella* are opportunistic pathogens that can cause severe diseases, such as sepsis, pneumonia, and other nosocomial infections (49). Hospitalized immunocompromised patients with underlying diseases are the main targets of these bacteria, so *Klebsiella* infections may serve as a model for hospital-acquired infections (50). In this cohort of ICU patients with AGI in the current study, *E. coli* was significantly associated with in-hospital mortality at the species level, thereby indicating its key microbial role in the progression of disease. *Klebsiella-*related shotgun metagenomic sequencing indicated that *K. pneumonia* and *K. variicola* were the main differential species in the present study. *K. pneumoniae infection* is usually related to a high incidence rate, while *K. pneumoniae* bacteremia is an important cause of high mortality in the general wards and the ICU (51). As a member of the *K. pneumoniae* complex, *K. variicola* was identified as a cause of several human infections and a significant risk factor for 30-day mortality in a single-center study (52, 53). These results enhanced the understanding of the interaction between gut microbiota and the risk of in-hospital mortality.

*Bacteroides* was identified in this study as the genus with the most abundant but the greatest variation, which seemed correlated with disease severity due to the relatively lower abundance in the Death group. Due to the introduction and excessive growth of competing microorganisms, the depletion of *Bacteroides* might represent a dysbiotic state in gut microbiota (54). Furthermore, based on the LEfSe analysis and Welch’s *t*-test results, the relative abundances of intestinal species *F. prausnitzii* and *P. copri* were markedly higher in the Survival group than in the Death group. *F. prausnitzii* is a well-known butyrate-producing bacteria in the GI tract of healthy human adults and has anti-inflammatory properties as a commensal bacterium (55). Thus, changes in the abundance of *F. prausnitzii* can be associated with metabolic and intestinal diseases in humans, such as IBD and chronic kidney disease (56). Meanwhile, *P. copri* has been used as an indicator of favorable cardiometabolic and postprandial glucose markers, which can potentially stratify health levels in individuals without clinically manifest disease (57). Further research on targeted intervention and regulation of gut microbiota would be more helpful in explaining the findings.

Along with the alteration in the composition of gut microbiota, the functional pathways also underwent corresponding dysregulation based on the results of this study. The super pathway of glycolysis, pyruvate dehydrogenase, TCA cycle I, and partial TCA cycle predominated in the Death group, while the biosynthesis pathways of L-histidine, UMP, flavin, and L-rhamnose were substantially enriched in the Survival group. In fact, both glucose metabolism and the TCA cycle can be regulated by the gut microbiota via microbe-derived metabolites, such as short-chain fatty acids and succinate (58, 59). The restriction of energy homeostasis seems to exacerbate the disease severity and hinder clinical outcomes for ICU patients (60). Specifically, *Klebsiella* might make significant contributions to altering pathways and disrupting functions. Furthermore, correlation analysis showed that *F. prausnitzii* was negatively correlated with the disease severity, indicating that the decrease in butyrate production may be closely associated with the disease severity.

As for the ARG features in this study, all the ICU patients with AGI received empirical antibiotics, so the ARGs exhibited more diversity and complexity, up to 27 types and 603 subtypes. In the Survival group, the ARG types of tetracycline and MLS had relatively higher abundances, and the top abundance of the *tetQ* subtype may reflect a higher abundance of *Bacteroidota*, as *tetQ* is widely distributed on the binding transposons in the phylum (61). Compared to the Survival group, the relative abundances of multidrug, other peptide antibiotics, aminoglycoside, fosfomycin, sulfonamide, and quinolone ARGs were significantly increased in the Death group, the same as the relative abundance of *mdtK*, *aph(3'')-Ib*, *microcin efflux pumu gene yojI*, *FosA family*, *sul1*, and *QnrB family* subtypes. For example, *sul1* can transfer between bacteria in different environments, and its high abundance indicates a high potential risk of horizontal gene transfer between different bacteria in the environment (62). Thus, these results reveal a potential correlation between the relative abundance of multidrug and aminoglycoside ARGs and the disease severity.

Our study found that the relative abundances of *FosA family*, *K. pneumoniae acrA*, and *aac(6’)-Ib-cr* subtypes in the Death group were positively related to *K. pneumoniae*, the most common carriers of ARGs (63). A study reported that the increase of *FosA family* profiles among Enterobacterales can be caused by the alteration of fosfomycin influx inside bacterial cells and acquiring antimicrobial resistance genes (64). Given that multidrug efflux is one of the resistant determinants in *K. pneumoniae*, *K. pneumoniae acrA* also became the significantly correlated ARG subtype. This property may result from the broadly specific drug efflux pumps, such as acrAB-TolC, which is the most important efflux pump among Gram-negative bacteria in clinical practice, thus attracting great attention as strategic targets for making bacteria re-sensitive to the existing antibiotics (65). As for the subtype of *aac(6’)-Ib-cr*, a plasmid-mediated quinolone/aminoglycoside resistance gene, this may refer to the drug modification due to *aac(6')-Ib-cr* acetyltransferase, which can facilitate to select higher-level resistance and raise a threat to the microbial community in treating infections (66). Our findings highlight that ICU patients with AGI, especially those in the Death group, may be susceptible to the accumulation of ARGs due to dysbiosis of gut microbiota, characterized by the enrichment of pathogenic bacteria such as *Klebsiella* (67).

Gut microbiota has been associated with in-hospital mortality of ICU patients, as well as the severity of symptoms and clinical outcomes (68). In the present study, the research results showed significant changes in the gut microbiota and ARG profile between the Death and Survival groups, indicating that they may become potential biomarkers for disease severity. Thus, the RF model was used to systematically compare the gut microbiota and ARGs as prognostic biomarkers for the disease severity. However, the AUC of 0.667 for the species classification model seemed relatively low (less than 0.7), suggesting that additional research is needed to expand on the possibility to predict the probability of survival on the basis of microbiome data. Fortunately, the results indicated that the ARG type showed better predictive performance than gut microbiota. The combination of gut microbiota and ARG subtypes could also increase performance for disease severity prediction than the gut microbiota or AGR subtype alone.

The major limitations of this study were the monocentric cohort and the relatively small sample size in both groups. In addition, the evaluation of variations among patients was considered, especially from different locations. Thus, individual patient demographic factors, such as underlying diseases, may have affected the results of the statistical analysis. Furthermore, only the correlation between microbiome/resistome changes and disease severity was defined, rather than a direct causal relationship with the disease severity according to the present results. Nevertheless, this study still provides valuable information on the gut microbiota and the risk of in-hospital mortality in ICU patients with AGI. Future studies on predicting in-hospital mortality may need stricter criteria in multicenter cohorts and more mechanistic investigations to confirm our results.

### Conclusions

A metagenomic-related strategy was conducted to obtain a highly valuable resource to improve understanding of intestinal microbiota dysbiosis and ARGs profiles. The results indicate that intestinal microbiota, including *Klebsiella* and *Prevotella*, changed dramatically in ICU patients with AGI. Due to longer ICU stays and receiving more antibiotic treatment, the types and correlations of ARGs in the Death group were significantly higher than those in the Survival group. The findings of this study are expected to expand our knowledge of gut microbiota and resistome profiles reflecting GI status, accelerate the identification of disease biomarkers, and provide new insights into the prevention and treatment of AGI-related diseases.

## Data Availability

The raw sequence data reported in this paper have been deposited in the Genome Sequence Archive in National Genomics Data Center (69), China National Center for Bioinformation/Beijing Institute of Genomics (70), and Chinese Academy of Sciences (Bioproject No.: PRJCA011331; GSA No.: CRA007908 and CRA008809) that are publicly accessible at https://ngdc.cncb.ac.cn/gsa.

## SUPPLEMENTAL MATERIAL

Supplemental Figures msystems.01444-24-s0001.pdf Figures S1 to S12.

Table S1 msystems.01444-24-s0002.xlsx Relative abundance of the fecal microbiota composition at different levels in 210 samples.

Table S2 msystems.01444-24-s0003.xlsx Variations of fecal bacterial functional pathways by LEfSe analysis and Welch's *t*-test.

Table S3-death group msystems.01444-24-s0004.xlsx Relative abundance of 9,891 antibiotic resistance genes in 210 samples.

Table S3-survival group msystems.01444-24-s0005.xlsx Relative abundance of 9,891 antibiotic resistance genes in 210 samples.

Table S4 msystems.01444-24-s0006.xlsx The ARG types and abundances in both groups.

Table S5 msystems.01444-24-s0007.xlsx The ARG subtypes and abundances in both groups.

ASM does not own the copyrights to Supplemental Material that may be linked to, or accessed through, an article. The authors have granted ASM a non-exclusive, world-wide license to publish the Supplemental Material files. Please contact the corresponding author directly for reuse.

## Note

We mistakenly added Footnote a and Footnote b in Table 1. Please help us remove these two footnote symbols. Thanks a lot for your help.
