# Supplementary material for: Comparative metagenome-associated analysis of gut microbiota and antibiotic resistance genes in acute gastrointestinal injury patients with the risk of in-hospital mortality
Source: mSystems. 2025 Feb 27;10(3):e01444-24. doi: 10.1128/msystems.01444-24 (PMC11915821; doi:10.1128/msystems.01444-24)

Supplementary Materials

Table S1. Relative abundance of the faecal microbiota composition at different levels in 210 samples.

Table S2. Variations of faecal bacterial functional pathways by LEfSe analysis and Welch's t-test between Death and Survival groups, *p_correct* <0.05.

Table S3. Relative abundance of 9891 antibiotic resistance genes in 210 samples.

Table S4. The ARG types and abundances in both groups.

Table S5. The ARG subtypes and abundances in both groups.

Figure Legends

Figure S1. Variations in the composition of the faecal microbiota and abundance correlation analysis between the Death and Survival groups. The top 10 abundance stacking maps at (A) the genus level, (B) the species level. Correlation analysis of top 10 genus in (C) the Death group, (D) the Survival group.

Figure S2. Comparison of α-diversity between the Death and Survival groups using the Shannon index. (A) At the family level; (B) at the genus level; (C) at the species level; (D) at the functional pathway level.

Figure S3. Comparison of β-diversity using principal coordinate analysis between the Death and Survival groups based on the Bray-Curtis matrix. (A) At the family level; (B) at the genus level; (C) at the species level; (D) at the functional pathway level.

Figure S4. Pathway differences between Death and Survival groups. (A) LEfSe analysis (LDA > 2.5, $p < 0.05$); (B) STAMP analysis ($p\_corrected < 0.05$).

Figure S5. Variations in the composition of the faecal microbiota between the Death and Survival groups, according to Welch's *t*-test $p < 0.05$. (A) At the family level, (B) at the genus level and (C) at the species level. Blue indicates the enriched flora in the Death group, and orange indicates the enriched flora in the Survival group.

Figure S6. Variations in the composition of the faecal microbiota in a more age-homogeneous subset (age from 30 to 70 years old, p > 0.05) with both groups using STAMP analysis. (A) At the family level, (B) at the genus level and (C) at the species level. Blue indicates the enriched flora in the Death group, and orange indicates the enriched flora in the Survival group.

Figure S7. Composition of ARGs and regulator genes in both groups. (A) The relative abundance of ARGs types; (B) resistance mechanism in Death Group; (C) resistance mechanism in Survival Group.

Figure S8. Differential comparison of antibiotic resistance genes (ARGs) between the Death and Survival groups. (A) α-diversity (Shannon index); (B) β-diversity (PCoA), $p = 0.001$.

Figure S9. Correlated analysis of enriched ARG types. (A) The Death Group; (B) The Survival Group.

Figure S10. Box plots show the relative abundances of ARG subtypes that showed significant differences between the two groups.

Figure S11. Diagnostic potential of gut microbial markers with the separate pre-filter process for each group. (A) The top 13 species in terms of importance; (B) ROC curve of species.

Figure S12. Confusion matrix as the additional performance metrics for random forest analysis. (A) The pre-filter process for all samples in both groups; (B) The separate pre-filter process for each group.

Figure S1

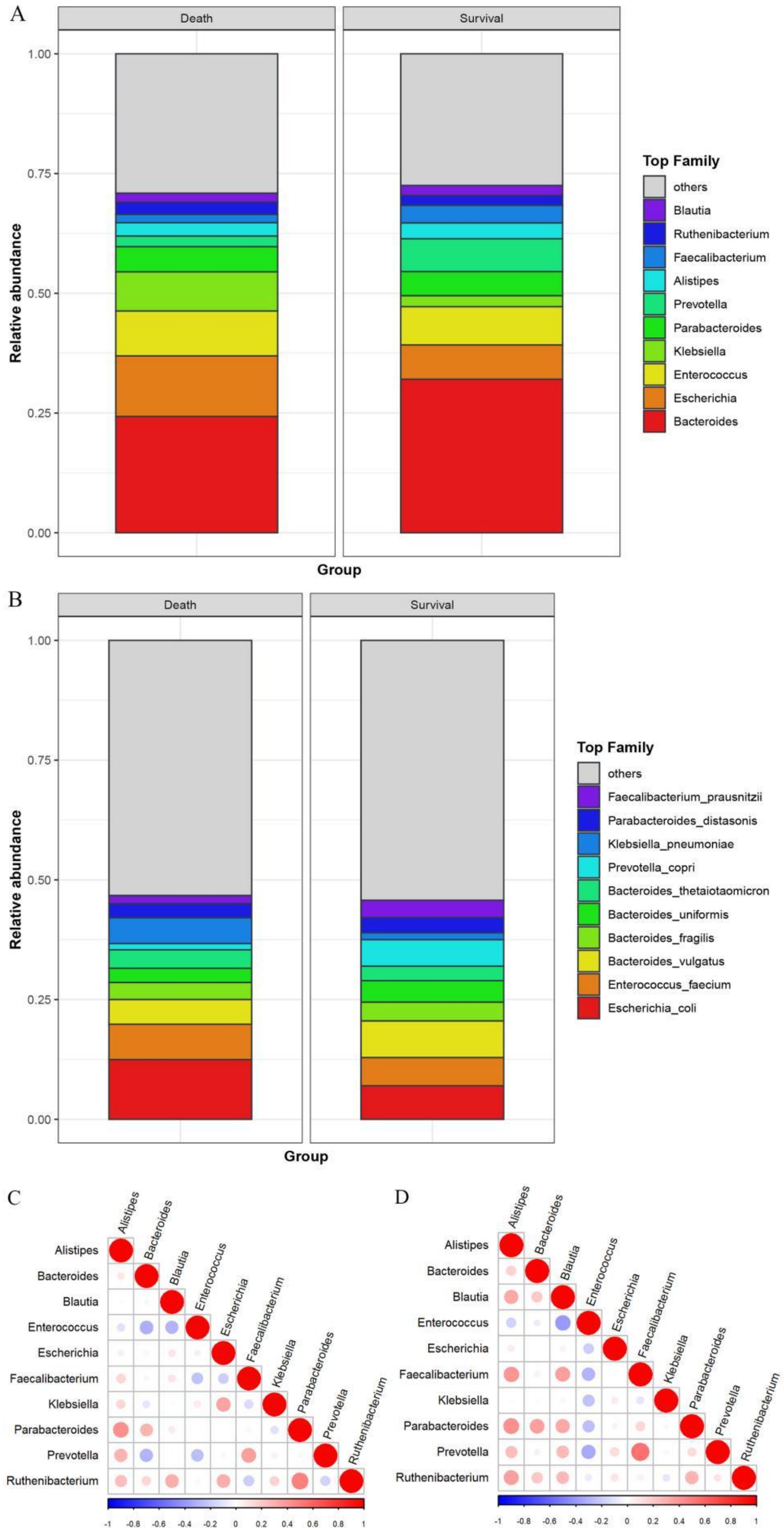
A
Death
Survival
Relative abundance
1.00
0.75
0.50
0.25
0.00
Group
Top Family
others
Blautia
Ruthenibacterium
Faecalibacterium
Alistipes
Prevotella
Parabacteroides
Klebsiella
Enterococcus
Escherichia
Bacteroides
B
Death
Survival
Relative abundance
1.00
0.75
0.50
0.25
0.00
Group
Top Family
others
Faecalibacterium_prausnitzii
Parabacteroides_distasonis
Klebsiella_pneumoniae
Prevotella_copri
Bacteroides_thetaiotaomicron
Bacteroides_uniformis
Bacteroides_fragilis
Bacteroides_vulgatus
Enterococcus_faecium
Escherichia_coli
C
Alistipes
Bacteroides
Blautia
Enterococcus
Escherichia
Faecalibacterium
Klebsiella
Parabacteroides
Prevotella
Ruthenibacterium
-1 -0.8 -0.6 -0.4 -0.2 0 0.2 0.4 0.6 0.8 1
D
Alistipes
Bacteroides
Blautia
Enterococcus
Escherichia
Faecalibacterium
Klebsiella
Parabacteroides
Prevotella
Ruthenibacterium
-1 -0.8 -0.6 -0.4 -0.2 0 0.2 0.4 0.6 0.8 1

Figure S2

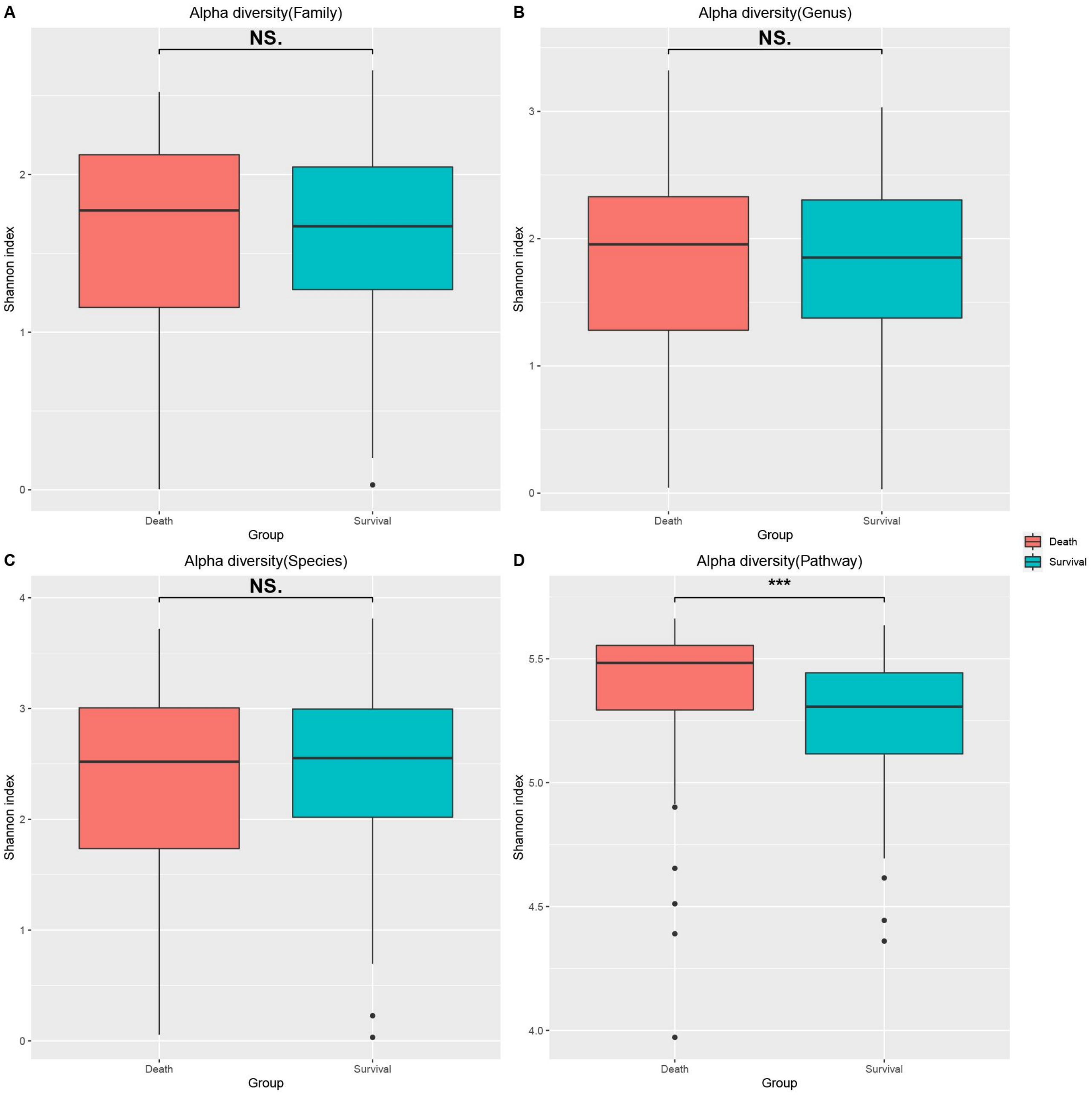

A
Alpha diversity(Family)
NS.
Shannon index
0
1
2
Death
Survival
Group
B
Alpha diversity(Genus)
NS.
Shannon index
0
1
2
3
Death
Survival
Group
C
Alpha diversity(Species)
NS.
Shannon index
0
1
2
3
4
Death
Survival
Group
D
Alpha diversity(Pathway)
***
Shannon index
4.0
4.5
5.0
5.5
Death
Survival
Group
Death
Survival

Figure S3

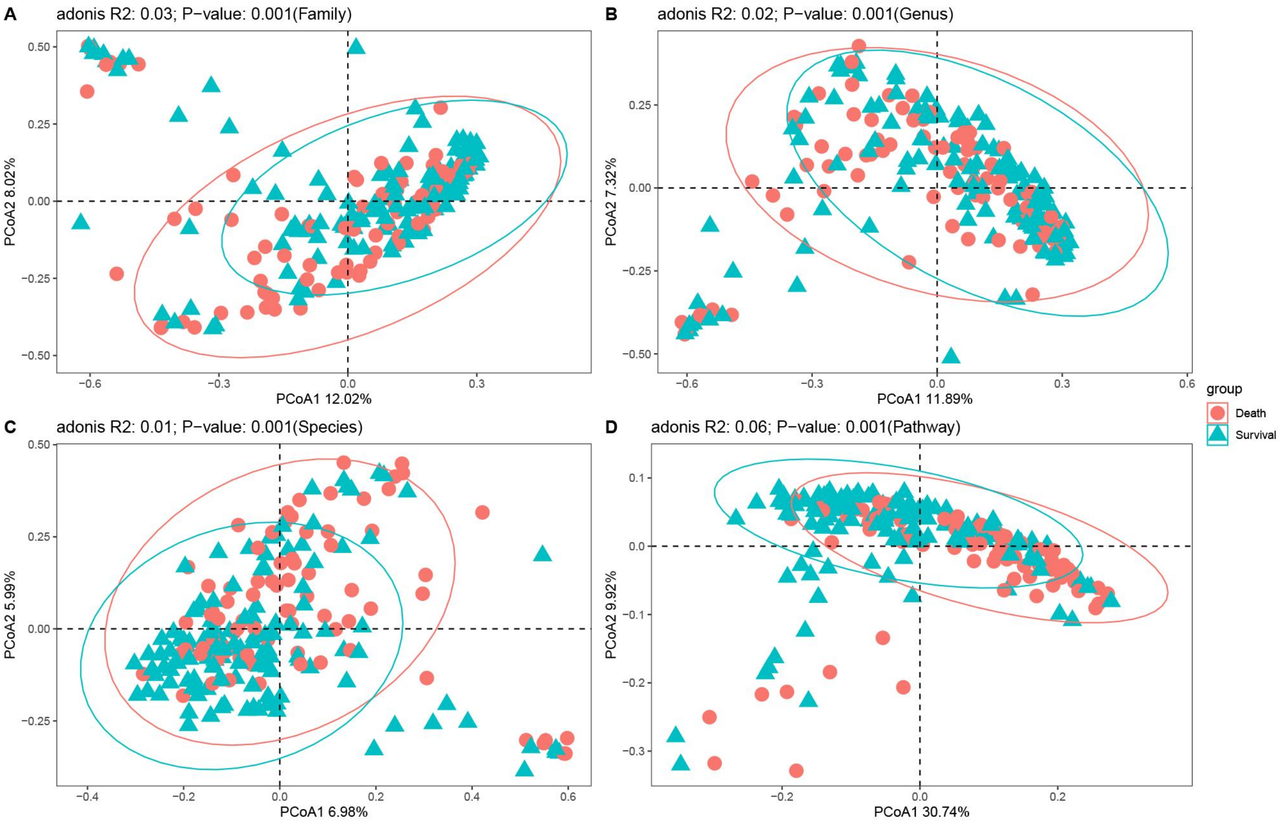

A
adonis R2: 0.03; P−value: 0.001(Family)
PCoA2 8.02%
PCoA1 12.02%
B
adonis R2: 0.02; P−value: 0.001(Genus)
PCoA2 7.32%
PCoA1 11.89%
C
adonis R2: 0.01; P−value: 0.001(Species)
PCoA2 5.99%
PCoA1 6.98%
D
adonis R2: 0.06; P−value: 0.001(Pathway)
PCoA2 9.92%
PCoA1 30.74%
group
Death
Survival

Figure S4

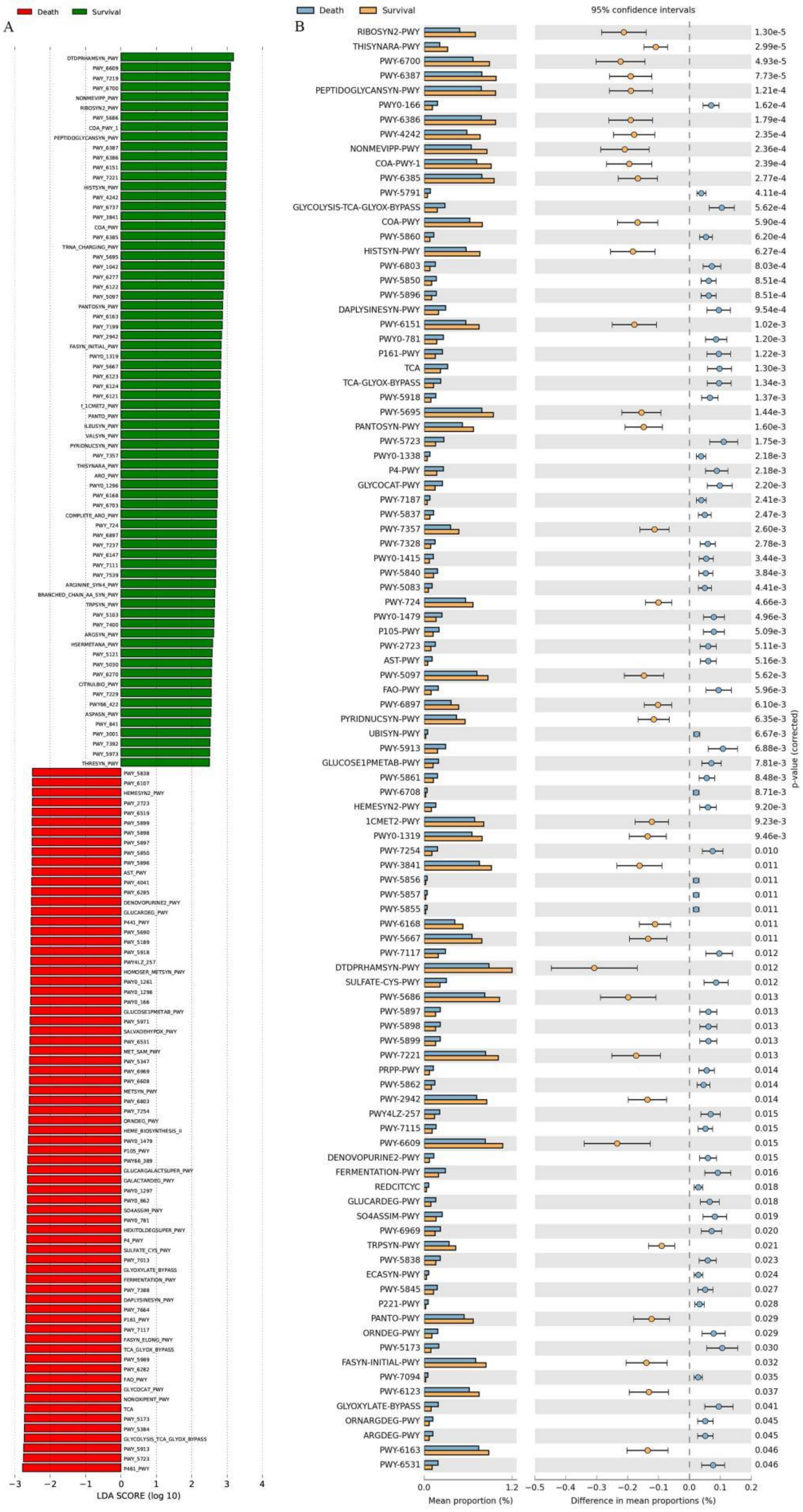
Death
Survival
A
LDA SCORE (log 10)
B
Death
Survival
95% confidence intervals
RIBOSYN2-PWY 1.30e-5
THISYNARA-PWY 2.99e-5
PWY-6700 4.93e-5
PWY-6387 7.73e-5
PEPTIDOGLYCANSYN-PWY 1.21e-4
PWY0-166 1.62e-4
PWY-6386 1.79e-4
PWY-4242 2.35e-4
NONMEVIPP-PWY 2.36e-4
COA-PWY-1 2.39e-4
PWY-6385 2.77e-4
PWY-5791 4.11e-4
GLYCOLYSIS-TCA-GLYOX-BYPASS 5.62e-4
COA-PWY 5.90e-4
PWY-5860 6.20e-4
HISTSYN-PWY 6.27e-4
PWY-6803 8.03e-4
PWY-5850 8.51e-4
PWY-5896 8.51e-4
DAPLYSINESYN-PWY 9.54e-4
PWY-6151 1.02e-3
PWY0-781 1.20e-3
P161-PWY 1.22e-3
TCA 1.30e-3
TCA-GLYOX-BYPASS 1.34e-3
PWY-5918 1.37e-3
PWY-5695 1.44e-3
PANTOSYN-PWY 1.60e-3
PWY-5723 1.75e-3
PWY0-1338 2.18e-3
P4-PWY 2.18e-3
GLYCOCAT-PWY 2.20e-3
PWY-7187 2.41e-3
PWY-5837 2.47e-3
PWY-7357 2.60e-3
PWY-7328 2.78e-3
PWY0-1415 3.44e-3
PWY-5840 3.84e-3
PWY-5083 4.41e-3
PWY-724 4.66e-3
PWY0-1479 4.96e-3
P105-PWY 5.09e-3
PWY-2723 5.11e-3
AST-PWY 5.16e-3
PWY-5097 5.62e-3
FAO-PWY 5.96e-3
PWY-6897 6.10e-3
PYRIDNUCSYN-PWY 6.35e-3
UBISYN-PWY 6.67e-3
PWY-5913 6.88e-3
GLUCOSE1PMETAB-PWY 7.81e-3
PWY-5861 8.48e-3
PWY-6708 8.71e-3
HEMESYN2-PWY 9.20e-3
1CMET2-PWY 9.23e-3
PWY0-1319 9.46e-3
PWY-7254 0.010
PWY-3841 0.011
PWY-5856 0.011
PWY-5857 0.011
PWY-5855 0.011
PWY-6168 0.011
PWY-5667 0.011
PWY-7117 0.012
DTDPRHAMSYN-PWY 0.012
SULFATE-CYS-PWY 0.012
PWY-5686 0.013
PWY-5897 0.013
PWY-5898 0.013
PWY-5899 0.013
PWY-7221 0.013
PRPP-PWY 0.014
PWY-5862 0.014
PWY-2942 0.014
PWY4LZ-257 0.015
PWY-7115 0.015
PWY-6609 0.015
DENOVOPURINE2-PWY 0.015
FERMENTATION-PWY 0.016
REDCITCYC 0.018
GLUCARDEG-PWY 0.018
SO4ASSIM-PWY 0.019
PWY-6969 0.020
TRPSYN-PWY 0.021
PWY-5838 0.023
ECASYN-PWY 0.024
PWY-5845 0.027
P221-PWY 0.028
PANTO-PWY 0.029
ORNDEG-PWY 0.029
PWY-5173 0.030
FASYN-INITIAL-PWY 0.032
PWY-7094 0.035
PWY-6123 0.037
GLYOXYLATE-BYPASS 0.041
ORNARGDEG-PWY 0.045
ARGDEG-PWY 0.045
PWY-6163 0.046
PWY-6531 0.046
p-value (corrected)
Mean proportion (%)
Difference in mean proportions (%)

Figure S5

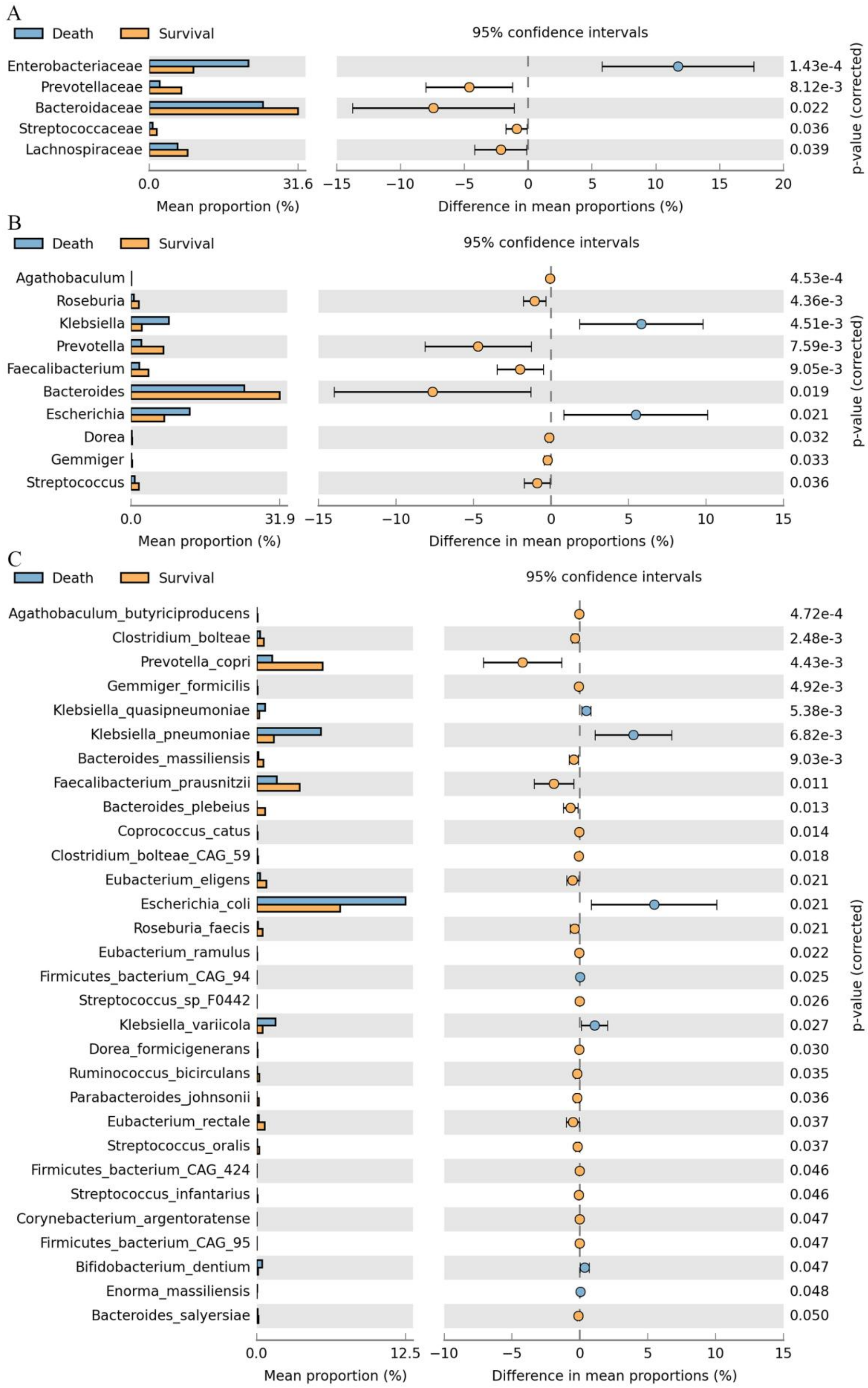

A
Death
Survival
95% confidence intervals
Enterobacteriaceae
Prevotellaceae
Bacteroidaceae
Streptococcaceae
Lachnospiraceae
1.43e-4
8.12e-3
0.022
0.036
0.039
p-value (corrected)
0.0
31.6
Mean proportion (%)
Difference in mean proportions (%)
B
Death
Survival
95% confidence intervals
Agathobaculum
Roseburia
Klebsiella
Prevotella
Faecalibacterium
Bacteroides
Escherichia
Dorea
Gemmiger
Streptococcus
4.53e-4
4.36e-3
4.51e-3
7.59e-3
9.05e-3
0.019
0.021
0.032
0.033
0.036
p-value (corrected)
0.0
31.9
Mean proportion (%)
Difference in mean proportions (%)
C
Death
Survival
95% confidence intervals
Agathobaculum_butyriciproducens
Clostridium_bolteae
Prevotella_copri
Gemmiger_formicilis
Klebsiella_quasipneumoniae
Klebsiella_pneumoniae
Bacteroides_massiliensis
Faecalibacterium_prausnitzii
Bacteroides_plebeius
Coprococcus_catus
Clostridium_bolteae_CAG_59
Eubacterium_eligens
Escherichia_coli
Roseburia_faecis
Eubacterium_ramulus
Firmicutes_bacterium_CAG_94
Streptococcus_sp_F0442
Klebsiella_variicola
Dorea_formicigenerans
Ruminococcus_bicirculans
Parabacteroides_johnsonii
Eubacterium_rectale
Streptococcus_oralis
Firmicutes_bacterium_CAG_424
Streptococcus_infantarius
Corynebacterium_argentoratense
Firmicutes_bacterium_CAG_95
Bifidobacterium_dentium
Enorma_massiliensis
Bacteroides_salyersiae
4.72e-4
2.48e-3
4.43e-3
4.92e-3
5.38e-3
6.82e-3
9.03e-3
0.011
0.013
0.014
0.018
0.021
0.021
0.021
0.022
0.025
0.026
0.027
0.030
0.035
0.036
0.037
0.037
0.046
0.046
0.047
0.047
0.047
0.048
0.050
p-value (corrected)
0.0
12.5
Mean proportion (%)
Difference in mean proportions (%)

Figure S6

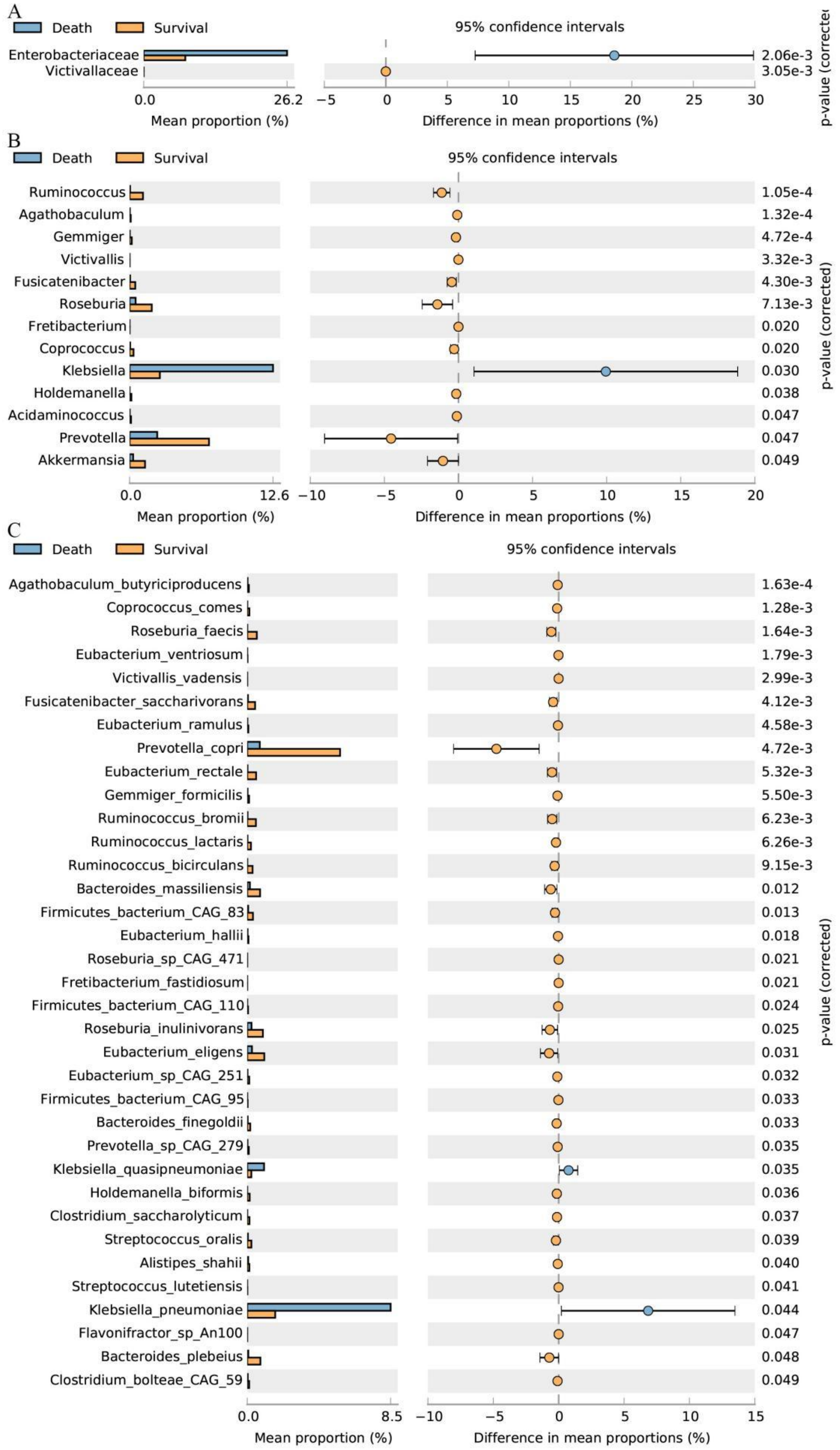
A
Death
Survival
95% confidence intervals
Enterobacteriaceae
Victivallaceae
2.06e-3
3.05e-3
Mean proportion (%)
Difference in mean proportions (%)
p-value (correcte
B
Death
Survival
95% confidence intervals
Ruminococcus 1.05e-4
Agathobaculum 1.32e-4
Gemmiger 4.72e-4
Victivallis 3.32e-3
Fusicatenibacter 4.30e-3
Roseburia 7.13e-3
Fretibacterium 0.020
Coprococcus 0.020
Klebsiella 0.030
Holdemanella 0.038
Acidaminococcus 0.047
Prevotella 0.047
Akkermansia 0.049
Mean proportion (%)
Difference in mean proportions (%)
p-value (corrected)
C
Death
Survival
95% confidence intervals
Agathobaculum_butyriciproducens 1.63e-4
Coprococcus_comes 1.28e-3
Roseburia_faecis 1.64e-3
Eubacterium_ventriosum 1.79e-3
Victivallis_vadensis 2.99e-3
Fusicatenibacter_saccharivorans 4.12e-3
Eubacterium_ramulus 4.58e-3
Prevotella_copri 4.72e-3
Eubacterium_rectale 5.32e-3
Gemmiger_formicilis 5.50e-3
Ruminococcus_bromii 6.23e-3
Ruminococcus_lactaris 6.26e-3
Ruminococcus_bicirculans 9.15e-3
Bacteroides_massiliensis 0.012
Firmicutes_bacterium_CAG_83 0.013
Eubacterium_hallii 0.018
Roseburia_sp_CAG_471 0.021
Fretibacterium_fastidiosum 0.021
Firmicutes_bacterium_CAG_110 0.024
Roseburia_inulinivorans 0.025
Eubacterium_eligens 0.031
Eubacterium_sp_CAG_251 0.032
Firmicutes_bacterium_CAG_95 0.033
Bacteroides_finegoldii 0.033
Prevotella_sp_CAG_279 0.035
Klebsiella_quasipneumoniae 0.035
Holdemanella_biformis 0.036
Clostridium_saccharolyticum 0.037
Streptococcus_oralis 0.039
Alistipes_shahii 0.040
Streptococcus_lutetiensis 0.041
Klebsiella_pneumoniae 0.044
Flavonifractor_sp_An100 0.047
Bacteroides_plebeius 0.048
Clostridium_bolteae_CAG_59 0.049
Mean proportion (%)
Difference in mean proportions (%)
p-value (corrected)

Figure S7

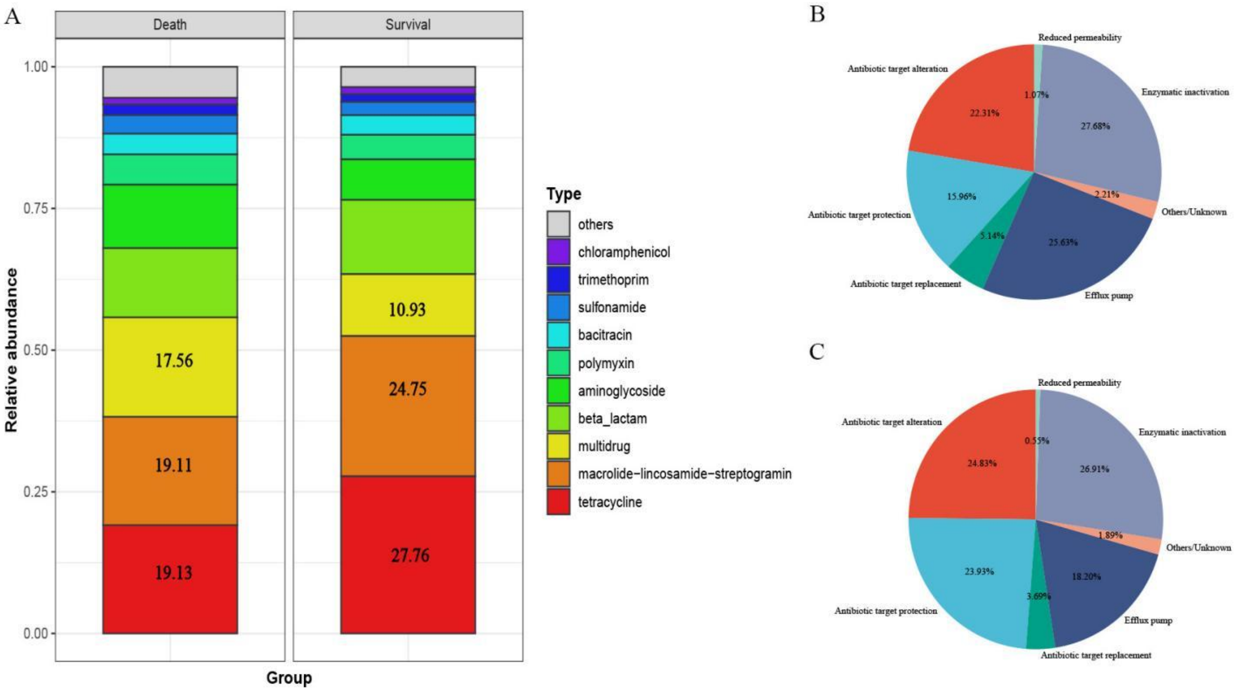

Figure S8

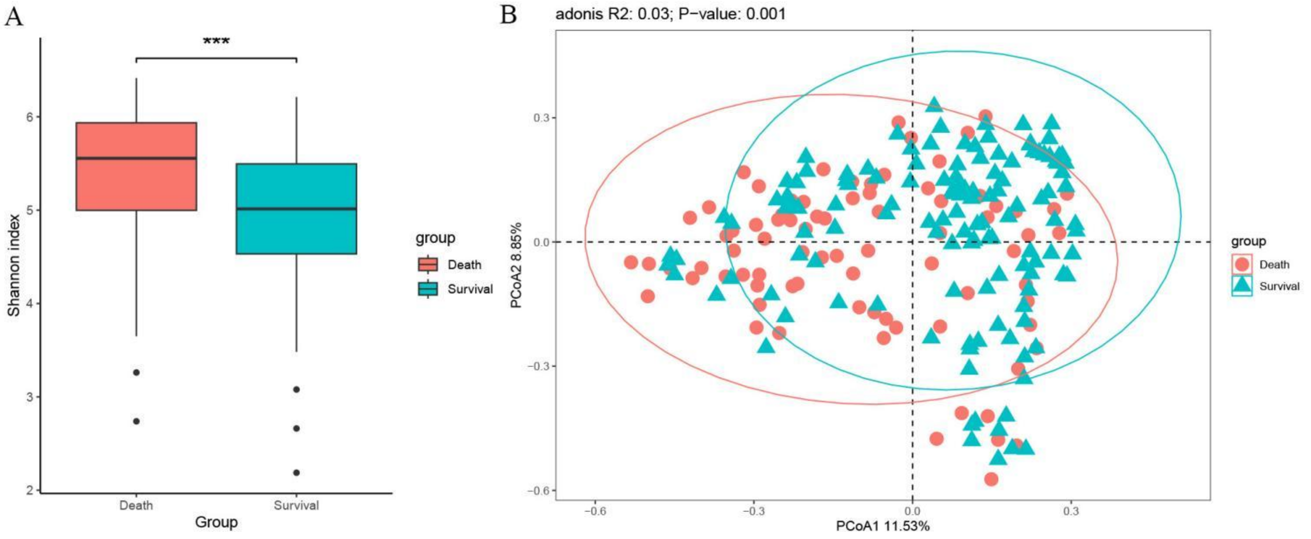

Figure S9

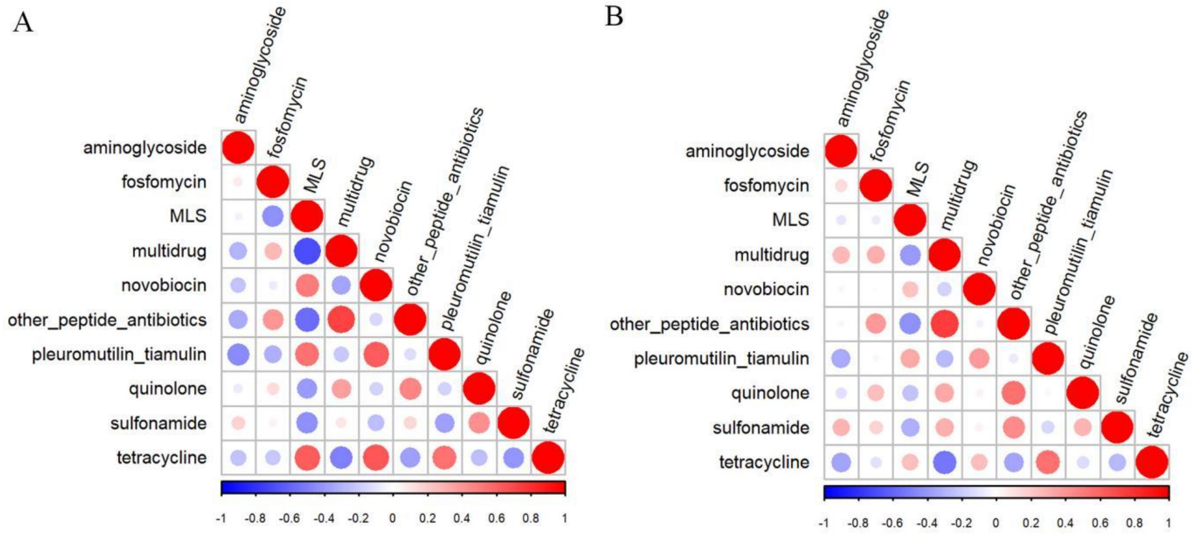
A
B
aminoglycoside
fosfomycin
MLS
multidrug
novobiocin
other_peptide_antibiotics
pleuromutilin_tiamulin
quinolone
sulfonamide
tetracycline
-1
-0.8
-0.6
-0.4
-0.2
0
0.2
0.4
0.6
0.8
1

Figure S10

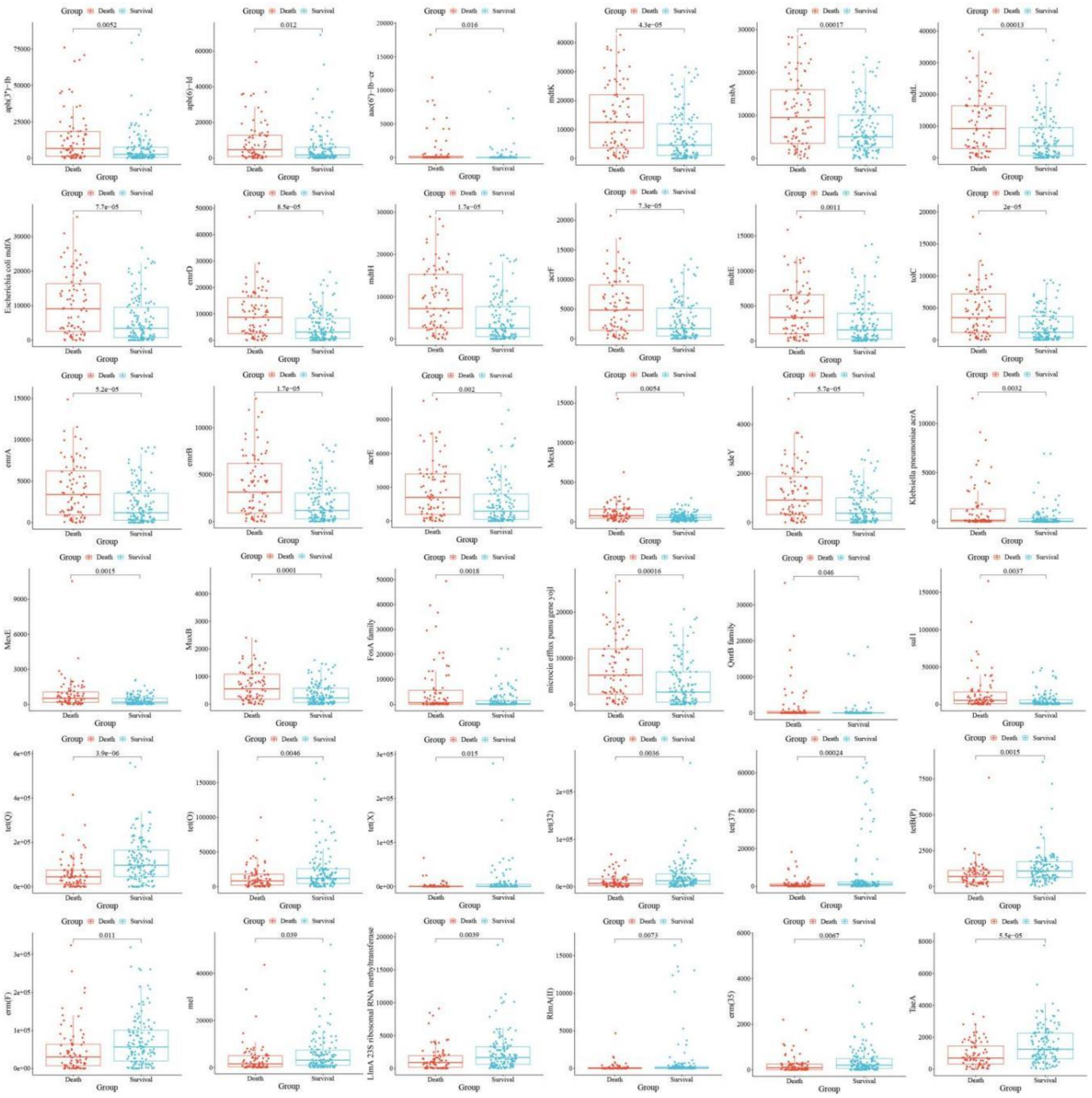

Group Death Survival
aph(3'')-Ib 0.0052
aph(6)-Id 0.012
aac(6')-Ib-cr 0.016
mdtK 4.3e-05
msbA 0.00017
mdtL 0.00013
Escherichia coli mdfA 7.7e-05
emrD 8.5e-05
mdtH 1.7e-05
acrF 7.3e-05
mdtE 0.0011
tolC 2e-05
emrA 5.2e-05
emrB 1.7e-05
acrE 0.002
MexB 0.0054
sdeY 5.7e-05
Klebsiella pneumoniae acrA 0.0032
MexE 0.0015
MuxB 0.0001
FosA family 0.0018
microcin efflux pump gene yojI 0.00016
QnrB family 0.046
sul1 0.0037
tet(Q) 3.9e-06
tet(O) 0.0046
tet(X) 0.015
tet(32) 0.0036
tet(37) 0.00024
tetB(P) 0.0015
erm(F) 0.011
mel 0.039
LlmA 23S ribosomal RNA methyltransferase 0.0039
RlmA(II) 0.0073
erm(35) 0.0067
TaeA 5.5e-05
Death
Survival
Group

Figure S11

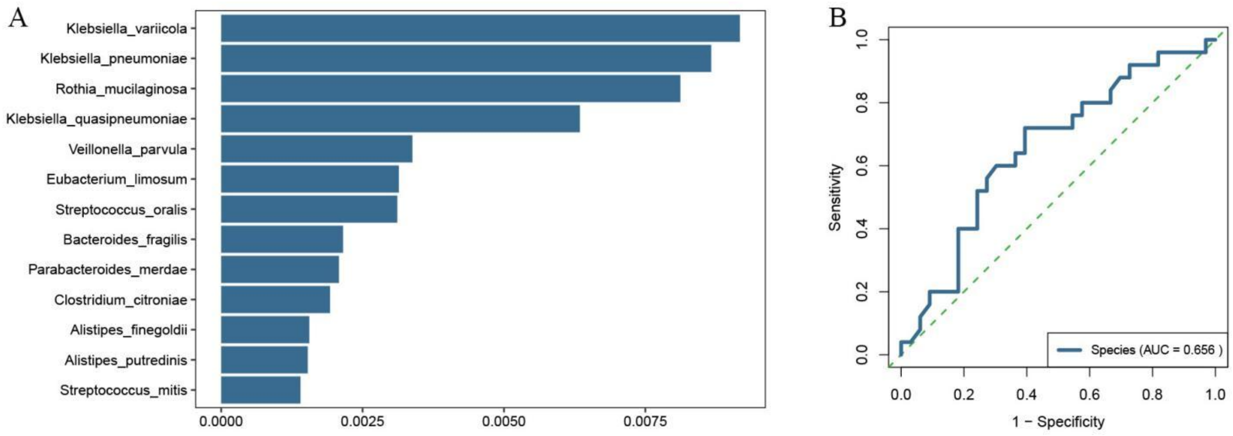

Figure S12

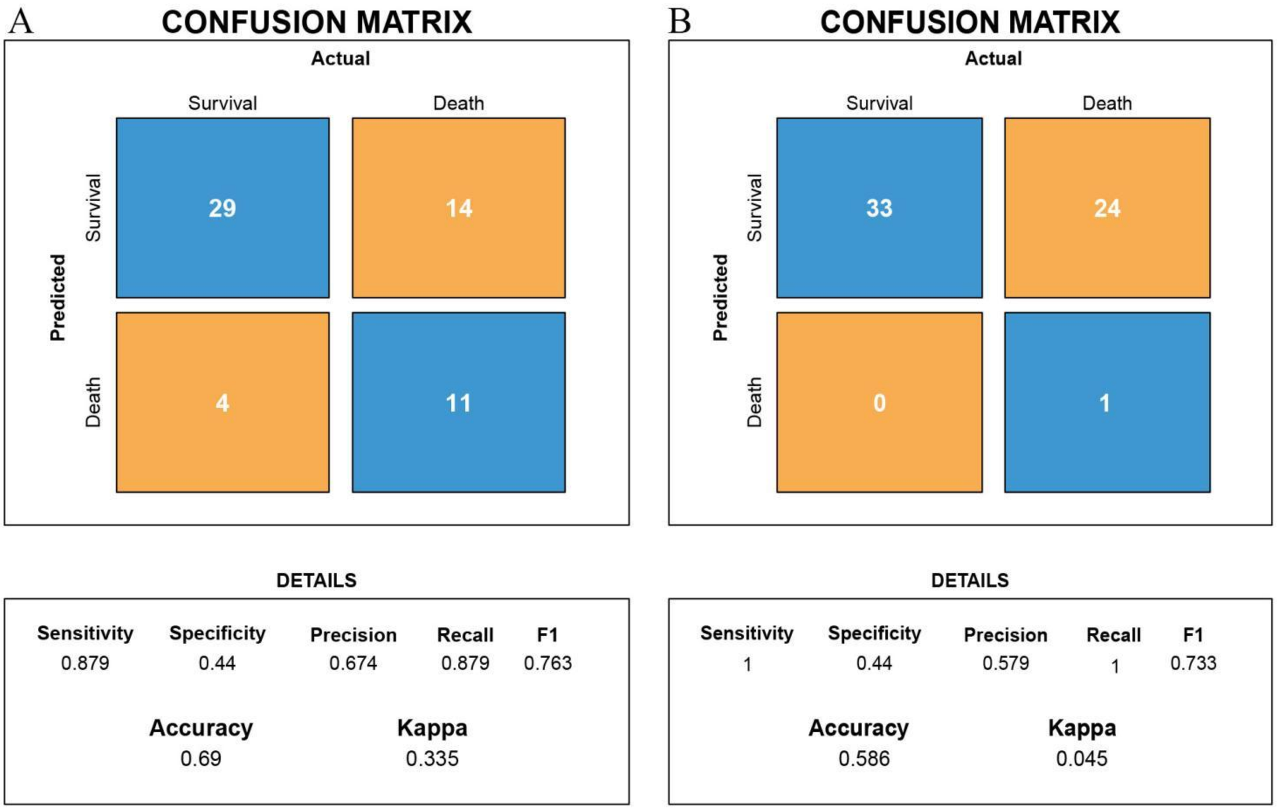

Supplement: Supplemental Figures — Figures S1 to S12. [file msystems.01444-24-s0001.pdf]
